# Supplementary material for: The impacts of climate change on hydrological processes of Gilgel Gibe catchment, southwest Ethiopia
Source: PLoS One. 2023 Jun 28;18(6):e0287314. doi: 10.1371/journal.pone.0287314 (PMC10306186; doi:10.1371/journal.pone.0287314)
Supplement: Attachment — Submitted filename: PONE-D-22-23861_reviewer_mod.pdf [file pone.0287314.s002.pdf]

PLOS ONE

# The Impacts of Climate Change on Hydrological Processes of Gilgel Gibe Catchment, Southwest Ethiopia

--Manuscript Draft--

| | |
|---|---|
| Manuscript Number: | PONE-D-22-23861 |
| Article Type: | Research Article |
| Full Title: | The Impacts of Climate Change on Hydrological Processes of Gilgel Gibe Catchment, Southwest Ethiopia |
| Short Title: | The Impacts of Climate Change on Hydrological Processes of Gilgel Gibe Catchment |
| Corresponding Author: | Zewde Alemayehu Tilahun, Ph.D. Student<br>Arba Minch University<br>Arba Minch, SNNPR ETHIOPIA |
| Keywords: | Climate change<br>Hydrological processes<br>SWAT model<br>RCP |
| Abstract: | Abstract:<br><br>Climate change affects the availability of water resources by altering the magnitude of surface runoff, aquifer recharge, and river flows. This study investigates the impact of climate change on hydrological processes of the Gilgel Gibe catchment. The assessment aids in determining how much the water resources are exposed to the change, which might be used to plan for future adaptability. For the future climatic scenarios in this study, the ensemble mean of six regional climate models (RCMs) from the coordinated regional climate downscaling experiment (CORDEX)-Africa was used. Distribution mapping was used to bias correct the RCMs outputs, with respect to the observed precipitation and temperature. Then, the Soil and Water Assessment Tool (SWAT) model was used to assess the hydrological impacts of climate change on the catchment. The result shows the ensemble mean of the six RCMs reported precipitation decline and increase in future temperature under both representative concentration pathways (RCP4.5 and RCP8.5). The increases in both maximum and minimum temperatures are higher for higher emission scenarios showing that RCP8.5 projection is warmer than RCP4.5. The changes in the projected climate change shows a decrease in surface runoff, groundwater and water yield. The overall decline of annual flow is due to the decline in the seasonal flows climate change scenarios. This could bring the reduced availability of water for crop production, which will be a chronic issue of subsistence agriculture. The possibility of surface water and groundwater reduction could also affect the availability of water resources in the catchment and further aggravate water stress in the downstream. The highly rising demands for water, owing to socio-economic progress and population growth as well as the variability in temperature and evaporation demands, amplify prolonged water scarcity. Consequently, strong climate-resilient water management policies will be indispensable to manage the risks. |
| Order of Authors: | Zewde Alemayehu Tilahun, Ph.D. Student |
| | Yechale Kebede Bizuneh, PhD (Associate Professor) |
| | Abren Gelaw Mekonnen, PhD (Assistance Professor) |
| Additional Information: | |
| Question | Response |
| **Financial Disclosure**<br><br>Enter a financial disclosure statement that describes the sources of funding for the work included in this submission. Review | The authors received no specific funding for this work. |

| | |
|---|---|
| the submission guidelines for detailed requirements. View published research articles from *PLOS ONE* for specific examples.<br><br>This statement is required for submission and **will appear in the published article** if the submission is accepted. Please make sure it is accurate.<br><br>**Unfunded studies**<br>Enter: *The author(s) received no specific funding for this work.*<br><br>**Funded studies**<br>Enter a statement with the following details:<br>• Initials of the authors who received each award<br>• Grant numbers awarded to each author<br>• The full name of each funder<br>• URL of each funder website<br>• Did the sponsors or funders play any role in the study design, data collection and analysis, decision to publish, or preparation of the manuscript?<br>• **NO** - Include this sentence at the end of your statement: *The funders had no role in study design, data collection and analysis, decision to publish, or preparation of the manuscript.*<br>• **YES** - Specify the role(s) played.<br><br>* typeset | |
| **Competing Interests**<br><br>Use the instructions below to enter a competing interest statement for this submission. On behalf of all authors, disclose any competing interests that could be perceived to bias this work—acknowledging all financial support and any other relevant financial or non-financial competing interests.<br><br>This statement is required for submission and **will appear in the published article** if the submission is accepted. Please make sure it is accurate and that any funding sources listed in your Funding Information later in the submission form are also | The authors have declared that no competing interests exist. |

| | |
|---|---|
| declared in your Financial Disclosure statement.<br><br>View published research articles from *PLOS ONE* for specific examples.<br><br>**NO authors have competing interests**<br><br>Enter: *The authors have declared that no competing interests exist.*<br><br>**Authors with competing interests**<br><br>Enter competing interest details beginning with this statement:<br><br>*I have read the journal's policy and the authors of this manuscript have the following competing interests: [insert competing interests here]*<br><br>* typeset | |
| **Ethics Statement**<br><br>Enter an ethics statement for this submission. This statement is required if the study involved:<br><br>• Human participants<br>• Human specimens or tissue<br>• Vertebrate animals or cephalopods<br>• Vertebrate embryos or tissues<br>• Field research<br><br>Write "N/A" if the submission does not require an ethics statement.<br><br>General guidance is provided below. Consult the submission guidelines for detailed instructions. **Make sure that all information entered here is included in the Methods section of the manuscript.** | N/A |

| | |
|---|---|
| **Format for specific study types**<br><br>**Human Subject Research (involving human participants and/or tissue)**<br>• Give the name of the institutional review board or ethics committee that approved the study<br>• Include the approval number and/or a statement indicating approval of this research<br>• Indicate the form of consent obtained (written/oral) or the reason that consent was not obtained (e.g. the data were analyzed anonymously)<br><br>**Animal Research (involving vertebrate animals, embryos or tissues)**<br>• Provide the name of the Institutional Animal Care and Use Committee (IACUC) or other relevant ethics board that reviewed the study protocol, and indicate whether they approved this research or granted a formal waiver of ethical approval<br>• Include an approval number if one was obtained<br>• If the study involved *non-human primates*, add *additional details* about animal welfare and steps taken to ameliorate suffering<br>• If anesthesia, euthanasia, or any kind of animal sacrifice is part of the study, include briefly which substances and/or methods were applied<br><br>**Field Research**<br><br>Include the following details if this study involves the collection of plant, animal, or other materials from a natural setting:<br>• Field permit number<br>• Name of the institution or relevant body that granted permission | |
| **Data Availability**<br><br>Authors are required to make all data underlying the findings described fully available, without restriction, and from the time of publication. PLOS allows rare exceptions to address legal and ethical concerns. See the PLOS Data Policy and FAQ for detailed information. | Yes - all data are fully available without restriction |

| | |
|---|---|
| A Data Availability Statement describing where the data can be found is required at submission. Your answers to this question constitute the Data Availability Statement and **will be published in the article**, if accepted.<br><br>**Important:** Stating 'data available on request from the author' is not sufficient. If your data are only available upon request, select 'No' for the first question and explain your exceptional situation in the text box.<br><br>Do the authors confirm that all data underlying the findings described in their manuscript are fully available without restriction? | |
| **Describe where the data may be found in full sentences. If you are copying our sample text, replace any instances of XXX with the appropriate details.**<br><br>• If the data are **held or will be held in a public repository**, include URLs, accession numbers or DOIs. If this information will only be available after acceptance, indicate this by ticking the box below. For example: *All XXX files are available from the XXX database (accession number(s) XXX, XXX.)*.<br>• If the data are all contained **within the manuscript and/or Supporting Information files**, enter the following: *All relevant data are within the manuscript and its Supporting Information files.*<br>• If neither of these applies but you are able to provide **details of access elsewhere**, with or without limitations, please do so. For example:<br><br>*Data cannot be shared publicly because of [XXX]. Data are available from the XXX Institutional Data Access / Ethics Committee (contact via XXX) for researchers who meet the criteria for access to confidential data.*<br><br>*The data underlying the results presented in the study are available from (include the name of the third party* | All relevant data are within the manuscript and its Supporting Information files. |

Powered by Editorial Manager® and ProduXion Manager® from Aries Systems Corporation

| | |
|---|---|
| *and contact information or URL).*<br>• This text is appropriate if the data are owned by a third party and authors do not have permission to share the data.<br><br>* typeset | |
| Additional data availability information: | Tick here if the URLs/accession numbers/DOIs will be available only after acceptance of the manuscript for publication so that we can ensure their inclusion before publication. |

Powered by Editorial Manager® and ProduXion Manager® from Aries Systems Corporation

Manuscript Click here to access/download;Manuscript;Manuscript witout Pics.docx

# THE IMPACTS OF CLIMATE CHANGE ON HYDROLOGICAL PROCESSES OF GILGEL GIBE CATCHMENT, SOUTHWEST ETHIOPIA

**BY**

**Zewde Alemayehu[1], Yechale Kebede[2], Abren Gelaw[3]**

[1]**Corresponding author:** PhD Candidate (Env't & Natural Resource management), Dep't of Geography & Env'tal Studies, Arba-Minch University, Ethiopia. Email: zedoalex8@gmail.com

[2]**Associate Professor** (Environmental Science), Dep't of Geography & Environmental Studies, Arba-Minch University, Ethiopia. Email: yechale.kebede@amu.edu.et

[3]**Assistance Professor** (Environment & Natural Resources Management), Dep't of Geography & Environmental Studies, Arba-Minch University, Ethiopia. Email: abren_mekon@yahoo.com

## *Abstract:*

*Climate change affects the availability of water resources by altering the magnitude of surface runoff, aquifer recharge, and river flows. This study investigates the impact of climate change on hydrological processes of the Gilgel Gibe catchment. The assessment aids in determining how much the water resources are exposed to the change, which might be used to plan for future adaptability. For the future climatic scenarios in this study, the ensemble mean of six regional climate models (RCMs) from the coordinated regional climate downscaling experiment (CORDEX)-Africa was used. Distribution mapping was used to bias correct the RCMs outputs, with respect to the observed precipitation and temperature. Then, the Soil and Water Assessment Tool (SWAT) model was used to assess the hydrological impacts of climate change on the catchment. The result shows the ensemble mean of the six RCMs reported precipitation decline and increase in future temperature under both representative concentration pathways (RCP4.5 and RCP8.5). The increases in both maximum and minimum temperatures are higher for higher emission scenarios showing that RCP8.5 projection is warmer than RCP4.5. The changes in the projected climate change shows a decrease in surface runoff, groundwater and water yield. The overall decline of annual flow is due to the decline in the seasonal flows climate change scenarios. This could bring the reduced availability of water for crop production, which will be a chronic issue of subsistence agriculture. The possibility of surface water and groundwater reduction could also affect the availability of water resources in the catchment and further aggravate water stress in the downstream. The highly rising demands for water, owing to socio-economic progress and population growth as well as the variability in temperature and evaporation demands, amplify prolonged water scarcity.*

*Consequently, strong climate-resilient water management policies will be indispensable to manage the risks.*

***Keywords:*** *Climate change, Hydrological processes, SWAT model, RCP*.

## 1. Introduction

The world environment is made up of numerous sophisticated systems that interact at different spatial and temporal scales, with impacts from both the natural and human worlds being deeply intertwined (Kasperson et al., 2022). The earth has been exposed to land degradation, which results in desertification and a decrease in land productivity due to conflicting interactions between past and present land use, socioeconomic activities, and ecological destructions (Shao et al., 2020).

Greenhouse gases (GHGs) concentrations in the atmosphere are thought to be rising as a result of human activity, particularly the combustion of fossil fuels and changes in LULC (Ribeiro et al., 2021). The change in the atmospheric gas composition tends to warm the atmosphere which will result in climate change (Al-Ghussain, 2019). Extreme hydrological events like floods and droughts are the primary causes of natural disaster in many parts of the world (Lindersson et al., 2020). The idea put forward by Clausius-Clapeyron theory states that, as a result of climate change, extreme incidences are likely to occur more frequently (Payne et al., 2011). Changes in precipitation and hydrological processes will also have a considerable impact on ecosystems, agricultural productions, and water resources (Shahvari et al., 2019).

Studies conducted in the past have demonstrated that the projected effects of climate change on hydrological processes vary significantly depending on the climate model and emission scenarios utilized in addition to observable spatial variance (Shiru et al., 2020; Kephe et al., 2021). Few studies have also attempted to evaluate the effects of climate change on hydrological process in Sub Saharan Africa, one of the most vulnerable region to climate change and climate variability (Bhaga et al., 2020). For the years 2046 to 2065 and 2081 to 2100, Roy et al. (2018) found a statistically significant increase in the Mara River flow volume at the Mara Mines river basin, Kenya/Tanzania. According to Getachew et al. (2021), the Gilgel Abay River in Ethiopia will experience an annual rise in flow volume from 2070 to 2100 as a result of climate change.

Over the study periods of the 2020s, 2050s, and 2080s, Hassaballah et al. (2019) also projected a rise in streamflow for the Dinder River in Sudan. However, Worku et al. (2021) suggested decline

in streamflow during mid 2040 to 2069 and late 2070 to 2099 for the Jemma sub-basin of upper Blue Nile Basin. Seasonal and yearly flow fluctuations, as well as flow declines in particular catchments, are primarily caused by erratic and unpredictable changes in climate factors (Radecki-Pawlik et al., 2020). In order to manage the water resource and environment in the face of future changes, scientific research is needed to understand the linkages between climate change and its effects. Further, new and updated insights on water and land conditions, as well as management options can facilitate more proactive approaches to maintain the water resources and land health through reversing degradation risk.

As the world's population grows, more water is being extracted to meet the demands of the agricultural, urban, industrial, and environmental sectors (Pérez et al., 2020). Therefore, a better understanding of land surface related hydrological processes under current climate conditions and future climate variability is required for adaptation to climate change and its effects. From these fundamental understandings, land and water management practices can then be developed.

A sufficient understanding of water resources development based on the projected climate is increasingly necessary for sustainable water resources management (Sheffield et al., 2018). Design of new water resource management strategies will have to deal with uncertainty related to a changing climate in addition to natural variability, which is reflected in conventional water planning systems (Herman et al., 2020).

Studies on the effects of climate change in Ethiopia on future seasonal and annual hydrological variables have revealed rising pressures on the availability of water resources (Daba and You, 2020; Gedefaw et al., 2019). In Ethiopia's Rift Valley, there has been a reported decrease in precipitation and an increase in temperature (Hundera and et al 2019).

Studies of the effects of climate change on watershed hydrology are becoming a basis to understand the implications and vulnerabilities of climate change and assisting in the development of sustainable water resource management strategies (Tesfahunegn and Gebru, 2020; Shu et al., 2018; Rickards et al, 2020; Yang et al., 2021). The implications of climate change on hydrology at the catchment level are, however, poorly understood. Gilgel Gibe catchment is intensively utilized and environmentally vulnerable (Fanta et al., 2022; Woldeab et al., 2022; Wedajo et al., 2022). Gibe river is used for generating hydropower energy and fresh water supply for small towns along its course (Aneseyee et al., 2022).

Gilge Gibe catchment, a part of Omo Gibe basin is among the watersheds facing the challenges. Rapid population growth, with the expansion of cultivation lands, coupled with rapid urban expansion in the catchment, have brought unprecedented impact on the catchment (Akalu, 2022). Furthermore, the high rising demands of water in Gelgel Gibe catchment, due to the socio-economic progress and high demand of water for cultivation, are increasing pressure on water resources (Duguma and Janssens, 2021). However, evaluation of the impact of climate change on hydrological processes at catchment level based on the new climate scenario is not investigated in the catchment. The Gilgel Gibe catchment in southwest Ethiopia is one notable example where little information exists regarding the influence of climate change on hydrological processes, even though the stream is a major tributary to the Gibe river. Consequently, studies that consider the effects of climate change of the specific area are required at regional and local scales. Specifically, this research has been initiated to investigate the impact of climate change on hydrological process by using ensemble mean of six regional climate models (RCMs) in the coordinated regional climate downscaling experiment (CORDEX)-Africa

The goal of this research is to investigate the impact of climate change on the hydrology of Gilgel Gibe catchment. Specifically, this study was aimed at: (1) assessing and modelling the climate change and its impacts (2) assessing the impact of future climate change on the hydrology in Gilgel Gibe catchment 3) exploring how the water resources are sensitive to the changes. The findings of these studies provide plausible insights on the vulnerability of the Gilgel Gibe catchment to climate change.

# 2. Study Area and Research Methods

## 2.1 Description of the Study Area

**Location:**

The study was conducted at Gilgel Gibe Catchment which is situated on the upstream of the large Omo Gibe basin in in Jimma zone of Oromia National Regional State, southwestern part of Ethiopia, and is located 260 km southwest of Addis Ababa; its geographical coordinates are approximately $7^0$36'0" - $8^0$0'0" N latitude and $36^0$36'0" - $37^0$34'0"E longitude (ArcGIS 10.5 Figure 1). The catchment covers an area of about 514,103.4 ha with an altitude that varies between 1,096 and 3,341 m above sea level. The catchment covers Sekoru, Tiro Afeta, Kersa, Seka Chekorsa,

Omo Nada, Dedo, Shebe Sembo and Chora districts of Jimma zone and Jimma special town. The Gilgel Gibe is the main river of the catchment and is cut by the Gilgel Gibe hydroelectric dam.

**Soil:**

The slope gradient varies from flatlands on the valley bottom to very steep slopes on the surrounding mountain ranges. According to FAO/UNESCO (1995), the dominant soils of Gilgel Gibe catchment include Chromic Vertisols, Chromic Vertisols/Eutric Fluvisols;Dystric Nitoso, Chromic Vertisols/Eutric Nitosols, Dystric Nitosols, Dystric Nitosols/Orthic Acrisols, Eutric Nitosols/Chromic Vertisols, Lithosols/Eutric Cambisols, Orthic Acrisols/Dystric nitosols and Pellic Vertisols.

**Climate:**

Despite its location in the tropical zone, Ethiopia has a diverse range of climates; several factors give the country a particular set of different climates. Temperature and precipitation patterns are influenced by altitude, rift valley, and the Inter-Tropical Convergence Zone (ITCZ) across the country. Minimum, maximum, and average temperatures range from below 10°C to above 40°C, with annual rainfall ranging from 100mm to 2,800mm depending on the location. Furthermore, rainfall patterns vary across the country in terms of time. In some parts of Ethiopia, the majority of precipitation is split between two rain seasons (Belg and Kiremt), whilst in others, there is just one (CSA, 2007). The average minimum and maximum temperatures are 11.5°C and 27.5°C, respectively, with 1,521 mm of precipitation on average. Precipitation can range from 1,300 mm in the lower reaches to 2,000 mm in the upper reaches of the basin (CSA, 2007). The average monthly rainfall and monthly maximum and minimum temperature data for the stations is as shown below.

## 2.2 Research Methods

### 2.2.1 General circulation model (GCM) data

Daily rainfall and temperature time series from runs with six different a new generations of GCMs were obtained from the coupled model intercomparison project phase 5 (CMIP5) database of intergovernmental panel on climate change (IPCC) data distribution center (http://cmip-pcmdi.llnl.gov/cmip5/). The details of the models are shown in Table 1. The GCMs data considered for this study is for the periods 1991–2021 as control simulations while for the future

simulations, two periods were considered: the intermediate (2041-2070) and far future (2071-2099) periods. Future scenarios considered for this study are the IPCC fifth assessment report (AR5) (IPCC, 2013). The new scenarios representative concentration pathways (RCPs) have four pathways (RCP 8.5, RCP 6, RCP 4.5 and RCP 2.6). In this study a moderate climate change scenario (RCP 4.5) and an extreme scenario (RCP 8.5) were implemented to detect the possible impact of climate change. Summary of RCMs projections was presented in Table 1.

Table 1 Description of the climate models (RCMS) from which data is obtained for this study

| No | RCMs | Model Center |
|---|---|---|
| 1 | CCLM4-8 | Climate Limited Area modelling Community (CLMcom), USA |
| 2 | HIRHAM5 | Denmarks Meteorologiske institute (DMI), Denmark |
| 3 | RACMO22T | Koninklijk Nederlands Meteorologisch Instituut (KNMI), Netherlands |
| 4 | RCA4 | Sveriges Meteorologiska och Hydrologiska institute (SMHI), Sweden |
| 5 | CRCM5 | Universite du Quebec a Montreal (UQAM), Canada |
| 6 | REMO2009 | Max Planck Institute for Meteorology-Climate Service centre (MPI-CSC), Germany |
| | Ensemble | Ensemble mean of all RCMs |

### 2.2.3 Climate Change Scenarios

After evaluating the performance of Regional Climate Models (RCMs) in Coordinated Regional Climate Downscaling Experiment (CORDEX) Africa, six RCMs showing a better performance were selected. A similar study by Behulu et al. (2018) in the central Rift valley basin showed that the ensemble mean of five RCMs have shown better performance than individual RCMs. Using multi-RCMs for climate modelling helps to minimize uncertainties, compared to the use of a single RCMs. However, despite the reasonable performance of the RCMs including the ensemble mean, all RCMs have exhibited noticeable biases. Therefore, corrections to the biases should be done before the climate change impact study.

The RCMs used in this study were: CCLM4-8, HIRHAM5, RACMO22T, RCA4, CRCM5 and REMO2009 fed by data of the two representative concentration pathways (RCP) scenarios high emission scenario (RCP8.5) and mid-range mitigation emission (RCP4.5). The intermediate (2041-2070) and far future (2071-2099) periods served as future scenario periods and the period from 1991 to 2021 as a historical baseline to evaluate the climate changes. Other weather variables, including solar radiation, relative humidity and wind speed in baseline/historical period were considered in the future scenarios without making any change as the changes in these variables may not have a significant impact in modelling the climate change scenarios on local hydrology.

### 2.2.4 Bias Correction

The climate model data for hydrological modelling (CMhyd) (Rathjens et al., 2016), obtained from https: //swat.tamu. edu/software/, was used to process the precipitation and temperature bias correction. Chen et al. (2019) have provided a full review of the bias correction techniques. According to Chen et al. (2019) all the bias correction techniques have improved the simulation of precipitation and temperature. However, there are differences between the correction methods in the daily precipitation series, standard deviations, and percentiles. Based on mean absolute error ranking, distribution mapping was ranked both, for temperature and precipitation corrections.

The distribution mapping (DM) uses a transfer function to adjust the cumulative distribution of estimated data to the cumulative distribution of rain gauges (Teutschbein and Seibert, 2012). Luo et al. (2018) applied seven precipitation bias correction methods and five methods for temperature. The results showed that distribution mapping reproduces precipitation and temperature very well. A similar study by Zhang et al. (2018) compared five bias correction methods, using CMhyd, and found distribution mapping performed best for climate change impact study on streamflow dynamics of two rivers in Northern lake Erie basin, Canada. The study by Geleta and Gobosho (2018) used CMhyd for extraction of CORDEX-NetCDF, and bias correction of minimum and maximum temperature to predict climate change-induced temperature changes in Finchaa catchment. The study showed that distribution mapping was better in improving the simulation. Owing to all the above findings, distribution mapping served as a basis for the precipitation and temperature corrections before using CORDEX-RCMs outputs for a climate impact study.

### 2.3 Hydrological Modelling

The hydrological modelling component of this study used Soil and Water Assessment Tool (SWAT), Soil and Water Assessment Tool (SWAT) is a physically-based semi-distributed model that operates on a continues time scale (Arnold et al., 1998). SWAT model operates on a daily time step and predicts the impact of land management in large complex watersheds with varying soils, land use and management conditions. Major model components include DEM, weather, hydrology, soil and properties and land management (Neitsch et al., 2011). In SWAT, a watershed is divided into multiple sub-watersheds, which are then further subdivided into Hydrologic Response Units (HRUs) that comprise homogeneous land use, slope and soil characteristics. The SWAT interface used an ArcSWAT, a SWAT application within ArcGIS. The hydrologic cycle as simulated by SWAT is based on the water balance equation as developed by Neitsch et al. (2011):

$$S_{wt} = \mathrm{S_{wo}} + \sum_{i=1}^{t} \left(\mathrm{R_{day}} - \mathrm{Q_{surf}} - \mathrm{E_a} - \mathrm{W_{seep}} - \mathrm{Q_{gw}}\right) \ldots\ldots\ldots\ldots\ldots\ldots\ldots\ldots\ldots (1)$$

Where $S_{wt}$ is the final soil water content (mm), $\mathrm{S_{wo}}$ is the initial soil water content (mm), t is time (days), $\mathrm{R_{day}}$ is the amount of precipitation on day i (mm), $\mathrm{Q_{surf}}$ is the surface runoff on day i (mm), $\mathrm{E_a}$ is the amount of evapotranspiration on day i (mm), $\mathrm{W_{seep}}$ is the amount of percolation and bypass flow exiting the soil profile bottom on day i (mm), and $\mathrm{Q_{gw}}$ is the amount of return flow on day i (mm).

The ArcSWAT evolved from ArcSWAT2012 is an ArcGIS extension developed for an earlier version of SWAT. It provides a graphical user interface that allows for GIS data to be easily formatted for use in SWAT model simulations. In the SWAT model, the simulation of the hydrological process begins with watershed delineation and generating streamflow networks. With a 30 m resolution DEM, Gilgel Gibe catchment is delineated into 23 sub-watersheds and a multiple HRU was defined with classes of 10% land use, 20% soil and 10% slope. Accordingly, 428 HRUs were created. Then, with the input of weather data from nine stations (Sekoru, Jimma, Chora, Dedo, Shebe, Omo Nada, Kersa, Seka and Tiro Afeta), the SWAT model setup was ready for the first simulation, which was used for model evaluation. Sekoru and Jimma stations, consisting of all the climate variables, was considered a weather generator.

Before using the output of the SWAT simulation for analysis, the performance of the model was evaluated for the catchment. It is important to understand the conceptual framework of each step, as well as what data are used and how they are integrated into ArcSWAT. Figure 5 below shows the flowchart of modelling using ArcSWAT.

### .2.3.1 Model inputs

#### 2.3.1.1 Watershed

The Gilgel Gibe catchment digital elevation model (DEM) was extracted from the Shuttle Radar Topography Mission (SRTM) 30 meter resolution data from USGS website (https://earthexplorer .usgs.gov) Watershed boundary, sub-watersheds (SWs), hydrological response units (HRUs) and slope layers were defined using these DEM data. Delineation of the watershed has been made by considering the gauging station as an outlet. The watershed boundary was further divided into 23 sub-watersheds (SWs), which were then reapportioned into 428 HRUs to allow spatially detailed simulation by reflecting differences in various hydrological conditions for different LULC, soils and slope configurations (Figure 6).

#### 2.3.1.2 Land use and land cover

Landsat Thematic Mapper of 1991, Enhanced Thematic Mapper Plus of 2006, and Operational Land Imager or Thermal Infrared Sensor of 2021 were used for LULC classifications. The mosaic of Worldwide Reference System (WRS) Path 169, Row 54, Path 169, Row 55 and Path 170, Row 55 were used to extract the LULCs layers. Supervised image classification method recommended in Lillesand et al. (2015) and Campbell and Wynne (2011) were employed to interpret and classify the images.

#### 2.3.1.3 Soil

The SWAT model requires soil data to determine the hydrological parameters of each soil category within each SW and HRU. The major soil physicochemical characteristics such as soil depth, soil hydrological group, soil texture, bulk density, available water capacity, saturated hydraulic conductivity, organic carbon content, soil albedo, rock fragments and soil erodibility factors were considered in the analysis. The input soil layers in the ArcSWAT model were extracted from the

Food and Agriculture Organization of the United Nations (FAO) (FAO/UNESCO, 1995; Batjes, 1997) and Harmonized World Soil Database (HWSD) (Van et al, 2009).

### 2.3.1.4 Weather

Daily weather data (1991–2021) were used from nine precipitation and temperature recording stations located within and around the study Catchment (Sekoru 7° 55' 48.1188"N and 37° 25' 40.7388"E, Jimma 7° 40' 12.4788"N and 36° 49' 16.3812"E, Chora 8° 21' 0"N and 36° 7' 0.0012"E, Dedo 7° 30' 15.2388"N and 36° 52' 46.9812"E, Shebe 7° 30' 33.3612"N and 36° 31' 6.78"E, Omo Nada 7° 37' 48" N and 37° 15' 18" E, Kersa 7° 42' 0" N and 36° 58' 0.0012" E, Seka 7° 36' 0" N and 36° 43' 26.4" E, and Tiro Afeta 8° 1' 55.6212" N and 37° 12' 9.18" E) (Figure 7). The daily data were collected from the National Meteorological Agency (NMA) of Ethiopia. However, some missing weather elements such as precipitation, maximum and minimum temperature, wind speed, solar radiation and relative humidity data were generated using an ArcSWAT weather generator (WGEN). Moreover, weather data from 1991 and 1992 were used as the model initialization phase (warm up) and not included in the final analysis.

### 2.3.1.5 Discharge

The Gilgel Gibe catchment river discharge gauging stations, Daily discharge data of Gibe near Seka, Bulbul near Serbo, Gilgel Gibe at Abelti, Gilgel Gibe near Asendabo, Gojeb near Shebe, Bidru Awana near Sekoru, Kito near Jimma and Awaitu at Jimma gauging stations for the period 1991 to 2021 were collected Ministry of Water and Energy of Ethiopia (MoWE). These data were used for model calibration and validation. Model calibration was performed for the period from 1993 to 2011, whereas validation was performed for the period from 2012 to 2021.

### 2.3.1.6 Sensitivity Analysis, Calibration and Validation

Owing to a large number of flow parameters in SWAT, identifying the most sensitive parameters is necessary to improve the calibration of the hydrological model. Model calibration and validation was undertaken automatically using SWAT-CUP (Calibration and Uncertainty Programs). The most sensitive parameters that have a strong influence on the flow process were identified through the sensitivity analysis. The Sequential Uncertainty Fitting (SUFI-2) embedded in the SWAT-CUP

(Calibration and Uncertainty Program) was used to achieve the sensitivity analysis, calibration and validation (Abbaspour, 2015).

Calibration of the hydrological model is the process of estimating model parameters by comparing the model prediction with the observed data for the same condition (Abbaspour, 2015; Moriasi et al 2007). Calibrations are very critical for parameters that were not measured and are intrinsically heterogeneous and uncertain, as it serves to optimize the unknown model parameters. For model calibrations, rules of parameter regionalization given by Abbaspour et al. (2015) was used. Validation was used to test the calibrated model without further parameter adjustments with an independent dataset. Observed streamflow of 1991–2021 was split into a warm-up (1991–1992), calibration period (1993–2011) and validation period (2012–2021).

The fitness of the model simulation with the observed streamflow was expressed by statistics like coefficients of determination ($R^2$), Nash-Sutcliffe efficiency (NSE), percent bias (PBIAS) and the ratio of the root-mean-square error to the standard deviation of measured data (RSR). The model performance ratings were based on the statistics recommended by Moriasi et al. (2007) and Ayele et al. (2017) $R^2$ varies between 0 and 1, where higher value shows less error. NSE ranges from negative infinity to 1 (best). PBIAS close to 0 shows best the simulation, a negative value indicates overestimation and a positive value indicates under simulation of the model. RSR varies from zero to a large positive number. The lower RSR shows a better simulation of the model. Furthermore, for the sensitivity of each parameter t-test and p-values was considered, using a t-test (larger in absolute values are more sensitive), and p-values were used to determine the significance of the sensitivity (a value close to zero has more significance) (Abbaspour, 2015). Statistical values of $R^2$, NSE, PBIAS and RSR were computed using equations 2, 3, 4 and 5 respectively:

$$R^2 = \left\{ \frac{\sum_{i=1}^{n}(O_i - \bar{O})(P_i - \bar{P})}{[\sum_{i=1}^{n}(O_i - \bar{O})^2]^{0.5} [(\sum_{i=1}^{n}(P_i - \bar{P})^2]^{0.5}} \right\}^2 \ldots\ldots\ldots\ldots\ldots\ldots\ldots\ldots\ldots\ldots\ldots\ldots (2)$$

$$NSE = 1 - \left[ \frac{\sum_{i=1}^{n}(O_i - P_i)^2}{\sum_{i=1}^{n}(O_i - \bar{O})^2} \right] \ldots\ldots\ldots\ldots\ldots\ldots\ldots\ldots\ldots\ldots\ldots\ldots\ldots\ldots\ldots\ldots\ldots (3)$$

$$PBIAS = \left[ \frac{\sum_{i=1}^{n}(O_i - P_i)}{\sum_{i=1}^{n}(O_i)} * 100 \right] \ldots\ldots\ldots\ldots\ldots\ldots\ldots\ldots\ldots\ldots\ldots\ldots\ldots\ldots (4)$$

$$RSR = \frac{RMSE}{STDEV_{obs}} = \left[\frac{\sqrt{\sum_{i=1}^{n}(O_i - P_i)^2}}{\sqrt{\sum_{i=1}^{n}(O_i - \bar{O})^2}}\right] \quad (5)$$

Where $O_i$ is the $i^{th}$ observed value for the streamflow ($m^3/s$), and $P_i$ is the $i^{th}$ predicted value for the streamflow ($m^3/s$), $\bar{O}$ is the mean of observed streamflow for the entire evaluation time period ($m^3/s$), and $\bar{P}$ is the mean of model predicted stream flow for the entire evaluation time period ($m^3/s$), and $n$ is the total number of observations.

The propagation of uncertainties in model outputs in SUFI-2, expressed as the 95% probability distribution, calculated by the 2.5% and 97.5% levels of the cumulative distributions of output variables, is considered as 95PPU (Abbaspour, 2015). P-factor and R-factor statistics are considered quantifying, the fit between the result expressed as 95PPU and observation. P-factor, the percentage of observations covered by the 95PPU varies from 0 to 1 with the ideal value of 1 while for R-factor, the thickness of the 95PPU optimal value is around 1

# 3. Results and Discussion

## 3.1 Hydrological Model Performance

### 3.1.1 Sensitivity Analysis, Calibration and Validation

Sensitivity analysis for the simulated streamflow was performed using a daily observed flow to identify the most sensitive parameter with strong influence on model outputs. Initially, parameters related to surface runoff, groundwater, geomorphology, evaporation and soil water were considered and 9 parameters were identified as the most sensitive parameters for calibration. The given ranks are shown in Table 3. The calibration was carried outfitting the model to the streamflow record of 1993–2011 and validation was done by streamflow record of 2012-2021. Afterwards, the calibrated values of these 9 parameters were used for further simulations. The parameter ranking was taken from the last iterations of SUFI-2 based on t-stat and p-stat. The larger in the absolute value of t-stat and the smaller the p-value, the more sensitive is the parameter (Abbaspour, 2015). As it can be seen from the table, the top three most sensitive parameters are CN2 (moisture condition II curve number), SOL_AWC (Available water capacity of the soil layer) and RCHRG_DP (Deep aquifer percolation fraction) (Table 2).

**Table 2 Sensitivity analysis and calibrated parameters**

| No | Parameter Code | Parameter Description | Parameter Value Range | Sensitivity | | |
|---|---|---|---|---|---|---|
| | | | | t-stat | p-value | Sensitivity Rank |
| 1 | CN2 | Curve number II | -0.23 - 0.16 | - | 0.00 | 1 |
| 2 | SOL_AWC | Available soil water capacity (mm $H_2O$ $mm^{-1}$ soil) | 0 - 60.26 | -4.04 | 0.00 | 2 |
| 3 | RCHRG_DP | Deep aquifer percolation fraction | -0.04- 0.12 | -1.90 | 0.04 | 3 |
| 4 | CH_K2 | Effective hydraulic conductivity in main channel alluvium (mm $h^{-1}$) | 25.18-95.07 | -2.02 | 0.05 | 4 |
| 5 | ALPHA_BF | Base flow recession constant | 0.39- 1.08 | 1.61 | 0.07 | 5 |
| 6 | ESCO | Soil evaporation compensation factor | 0.84- 1.02 | -1.08 | 0.09 | 6 |
| 7 | CANMX | Maximum canopy storage (mm) | -0.04- 0.33 | -3.12 | 0.11 | 7 |
| 8 | SOL_BD | Moist bulk density ($Mg/m^3$) | 0.02- 1.06 | 1.38 | 0.13 | 8 |
| 9 | GW_REVAP | Ground water re-evaporation coefficient | 0.08- 0.69 | 2.3 | 0.19 | 9 |

The calibration results on mean monthly flow show that SWAT model is able to capture the observed streamflow with $R^2$, NSE, PBIAS and RSR of 0.76, 0.75, 1.02, and 0.49 respectively. The average monthly flow validation indicates $R^2$, NSE, PBIAS and RSR of 0.82, 0.77, 9.4 and 0.47 respectively. This indicates that the performance of the SWAT model in the validation is good enough to simulate the stream flow in the Gilgel Gibe catchment. Furthermore, p-factor and R-factor statistics showed good agreement with 0.87 and 0.79 during calibration and 0.82, and 0.76 during validation, respectively (Table 3). Overall, the statistics for goodness fit shows good agreement between the observed and simulated average monthly flow.

**Table 3 Summary statistics of calibration and uncertainty analysis of streamflow**

| No | | Objective function values (SUFI-2) | | | | | |
|---|---|---|---|---|---|---|---|
| | Method | P- factor | R-factor | $R^2$ | NSE | PBIAS | RSR |
| 1 | Calibration | 0.87 | 0.79 | 0.76 | 0.75 | 1.02 | 0.49 |

| | | | | | | | |
|---|---|---|---|---|---|---|---|
| 2 | Validation | 0.82 | 0.76 | 0.82 | 0.77 | 9.4 | 0.47 |

## 3.2 Climate Change Projections of the RCMs

The projection of each RCM individually as well as the ensemble mean of the RCMs were examined to show how well each model predicted temperature and precipitation. The distribution mapping of the bias-corrected precipitation and temperature for the future scenarios is compared to the baseline data sets (1991–2021). Further, the hydrological responses of the catchment are analyzed by quantifying and comparing the future water balance components (surface runoff, groundwater flow, total water yield and evapotranspiration).

### 3.2.1. Rainfall

The individual and ensemble mean of the six RCMs were used to assess climate change in two future scenarios; intermediate term (2041–2070) and far future term (2071–2099) with respect to the baseline period **(1991–2021)** under RCP4.5 and RCP8.5. The results of the projected precipitation by the individual RCMs show a different degree of precipitation changes. Except for HIRHAM5 in far future term, all the RCMs show precipitation decline under both scenarios. According to CCLM4-8, precipitation decreases by -15.87% under RCP4.5 in intermediate term and -30.67% under RCP8.5 scenarios in a far future term. In case of CRCM5, precipitation decreases by -11.66% under RCP4.5 in intermediate term and -10.22% under RCP8.5 scenarios in a far future term. regarding REMO2009, precipitation decreases by -9.96% under RCP4.5 in intermediate term and -4.81% under RCP8.5 scenarios in a far future term. The highest decline of the precipitation was simulated under RCP8.5 in a far future term with CCLM4-8 and the lowest decline by HIRHAM5 under RCP4.5 in a far future term. HIRHAM5 shows that precipitation will increase under RCP8.5 scenarios.

Table 4 Changes in precipitation (%) across the four RCMs in RCP4.5 and RCP8.5 scenarios

| **No** | **RCMs** | **RCP4.5** | | **RCP8.5** | |
|---|---|---|---|---|---|
| | | **2041–2070** | **2071–2099** | **2041–2070** | **2071–2099** |
| 1 | CCLM4-8 | -15.87 | -18.68 | -18.84 | -30.67 |
| 2 | HIRHAM5 | -6.61 | -4.09 | 2.37 | 7.62 |

| | | | | | |
|---|---|---|---|---|---|
| 3 | RACMO22T | -12.51 | -20.2 | -9.59 | -7.62 |
| 4 | RCA4 | -10.77 | -15.16 | -13.48 | -14.42 |
| 5 | CRCM5 | -11.66 | -14.32 | -8.69 | -10.22 |
| 6 | REMO2009 | -9.96 | -13.15 | -6.90 | -4.81 |
| | Ensemble | -11.23 | -14.27 | -9.19 | -10.02 |

The ensemble mean of the RCMs suggested decreasing precipitation under the two scenarios, like most of the RCMs. The change shows precipitation decline by -11.23% under RCP4.5 and -9.19% under RCP8.5 in the intermediate term scenario. Under RCP4.5 and RCP8.5, the decline in the far future was -14.27% and -10.02%, respectively. The use of ensemble mean over the individual RCMs helps to minimize the highest and lowest projections and enables to minimize the uncertainties by working with the average of the RCMs. Furthermore, the analysis of the inter-annular and inter-model variability of the climate variables in Upper Blue Nile Basin catchment, presented in Dibaba et al. (20919), underlines the need to include several climate models in order to cover the range of possible developments.

Changes in projected precipitation are more profound on seasonal bases compared to the annual bases in the catchment. The projected precipitation shows a declining trend in all seasons, except in April by RCP8.5, for both future time horizons and August by RCP4.5 in an intermediate term scenario. Figure 10 illustrates that the expected changes are higher in the dry season than a wet season. The seasons with lower rainfall (March, April, and May) are likely to have significant changes in precipitation, whereas the seasons with higher rainfall (June, July, August, and September) are expected to experience less change. All estimates show a higher declining signal for dry seasons.

Using data from 1965 to 2002, it was shown that the intensity of precipitation was decreasing in Ethiopia's eastern, southwestern, and southern areas (Muluneh et al., 2017). Various studies in the Blue Nile basin, however, reported varying changes in precipitation in the future periods. According to Worku et al. (2020), 10 out of the 17 GCMs reported a decrease in precipitation, whereas the ensemble of the 17 GCMs shows that there was essentially no projected change in precipitation. On the other hand, Mohammed et al. (2020) reported an increase in projected precipitation in the late 21st century using 11 GCMs. Mengistu et al. (2021) have also reported

significant RCM-GCM uncertainty on the signal of future precipitation in the upper Blue Nile (Abay) river basin. This may be due to the nature of precipitation and its dependence on topographical and physical elements.

The results of precipitation projections in this study are consistent with those of studies on climate change (Gadissa et al., 2018; Behulu et al., 2019). Decreased precipitation was also noted in the study of Daba (2018) on the effects of climate change on the hydro-meteorological variables of the Finchaa sub-basin using the HadCM GCM. The study by Gebre et al., (2015) which also used CCLM downscaling, showed falling precipitation by 6.6% and 6.4% over the upper Blue Nile basin for the periods 2041-2070 and 2071-2100.

### 3.2.2 Temperature

Table 5 shows the range in the predicted future maximum and minimum temperatures for each individual RCM and their ensemble mean. The result shows that maximum and minimum temperatures increase under both RCPs throughout the study years considered showing the warming trends in the catchment. However, compared to RCP4.5, the magnitude of changes made by each individual RCM and their mean ensemble is greater for higher emission scenarios (RCP8.5). Under both emission scenarios, the change in temperature is also greater in the far future than the intermediate period. The magnitude of the temperature changes varies across all RCMs and the ensemble mean as well. Under both RCPs for the intermediate and far future, RCA4 has shown the greatest increase in maximum temperature.

Table 5 Changes in temperature (◦C) across the four RCMs in RCP4.5 and RCP8.5 scenarios

| No | Tmax | RCP4.5 | | RCP8.5 | |
|---|---|---|---|---|---|
| | RCMs | 2041–2070 | 2071–2099 | 2041–2070 | 2071–2099 |
| 1 | CCLM4-8 | 1.61 | 2.36 | 1.89 | 3.54 |
| 2 | HIRHAM5 | 1.54 | 2.42 | 1.66 | 3.55 |
| 3 | RACMO22T | 1.78 | 2.55 | 1.77 | 3.35 |
| 4 | RCA4 | 1.84 | 2.65 | 2.06 | 3.82 |
| 5 | CRCM5 | 1.64 | 2.44 | 1.77 | 3.48 |
| 6 | REMO2009 | 1.72 | 2.54 | 1.83 | 3.57 |

| | | | | |
|---|---|---|---|---|
| Ensemble | 1.69 | 2.49 | 1.83 | 3.55 |

Table 6 Changes in temperature (◦C) across the four RCMs in RCP4.5 and RCP8.5 scenarios

| No | Tmin | RCP4.5 | | RCP8.5 | |
|---|---|---|---|---|---|
| | **RCMs** | **2041–2070** | **2071–2099** | **2041–2070** | **2071–2099** |
| 1 | CCLM4-8 | 1.98 | 3.31 | 2.45 | 5.21 |
| 2 | HIRHAM5 | 1.74 | 2.91 | 1.89 | 4.44 |
| 3 | RACMO22T | 1.58 | 2.59 | 1.94 | 4.05 |
| 4 | RCA4 | 1.52 | 2.38 | 1.93 | 3.73 |
| 5 | CRCM5 | 1.77 | 2.94 | 2.09 | 4.57 |
| 6 | REMO2009 | 1.61 | 2.63 | 1.92 | 4.07 |
| | Ensemble | 1.70 | 2.79 | 2.04 | 4.35 |

Concerning to the ensemble mean of the RCMs, under the medium emission scenario (RCP4.5), the maximum temperature would rise by an average of 1.69 $^{o}$C and 2.49 $^{o}$C, while the minimum temperature will rise by an average of 1.70 $^{o}$C and 2.79 $^{o}$C for the intermediate term and far future, respectively. Under high emission scenarios RCP8.5, the maximum temperature will rise by 1.83 $^{o}$C and 3.55 $^{o}$C, while the minimum temperature would rise by 2.04 $^{o}$C and 4.35 $^{o}$C, respectively, for the intermediate and far future (Table 6). Overall, the analysis showed that RCP8.5's estimate is warmer than RCP4.5's. Shiferaw et al. (2018) used five distinct GCMs which confirmed the maximum temperature change in RCP8.5 in the Ilala watershed, Northern Ethiopia. In addition to changing annually, the forecasted temperature changes were also anticipated to vary seasonally. Figure 11 illustrates that the seasonal changes are greater for the minimum temperature than the maximum temperature. Maximum temperature fluctuations are more pronounced during dry seasons (December, January, and February), while minimum temperature changes are more pronounced during wet seasons (June, July, and August). The highest seasonal rise of maximum temperature is reported by RCP8.5 during **2071–2099 while RCP 4.5 reported the lowest rise during 2041–2070.**

Similar to annual fluctuations, the high emission scenario is anticipated to cause a greater seasonal rise in temperature than the medium emission scenario. In addition, the far future scenario (2071-2099) is anticipated to have higher increases in maximum and minimum temperatures than the intermediate future scenario (2041–2070

Overall, the forecast of the maximum temperature and minimum temperature over both future time horizons are consistent with other researchers' ranges and falls within the IPCC's range. Numerous research on climate change in Ethiopia have suggested that temperatures are likely to rise across the country's various regions. However, the degree of change varies according on the downscaling methods and kinds of climate models. According to GFDRR's (2011) analysis on the country profile for climate risk and adaptation, the mean annual temperature is expected to rise by 1.1 to 3.1 degrees Celsius by the 2060s and 1.5 to 5.1 degrees Celsius by the 2090s. According to the analysis by Gebre et la., (2015), the entire upper Blue Nile basin's average annual temperature will rise by 1.5°C, 2.6°C, and 4.5°C between the years 2011 and 2040, 2041 and 2070, and 207 and 2100, respectively. According to Hasan et al (2018) study's on the entire Nile Basin, which used an average of 11 GCMs, the yearly average temperature change between 1950 and 1999 and 2010 to 2039 will range from 0.91 to 1.9 degrees Celsius. Furthermore, Mengistu et al (2021).'s study on the Blue Nile, which used 17 GCMs, predicted that temperatures will rise by 2 ˚C and 5 ˚C.

### 3.2.3 Impacts of Climate Change

Changes in rainfall and temperature were used to predict the impacts of climate change on water balance components of the catchment. Consequently, the projected changes in mean annual precipitation and temperature, under the two RCPs, caused a significant variation in the projected water balance components of Gilgel Gibe catchment.

Reduced surface flow, groundwater, and overall water yield are predicted as a result of the decline in precipitation and rise in temperature, according to SWAT models for the intermediate term and far future Table7. Changes in temperature and PET are correlated positively. In turn, this led to a probable rise in evapotranspiration and evaporation, which may have a significant impact on the decline in water yield. Evaporation is a factor in the future decline of the surface runoff since the forms of water are prone to losses due to temperature changes. This is expected considering the warming trends of temperature.

Table 7 Changes of water balance components under a climate change

| Scenario | Period | Surface Runoff (%) | Groundwater | Water Yield (%) | PET (%) |
|---|---|---|---|---|---|

| | | | flow (%) | | |
|---|---|---|---|---|---|
| RCP4.5 | 2041–2070 | -7.56 | -9.44 | -8.72 | 16.08 |
| | 2071–2099 | -14.71 | -13.93 | -14 | 19.01 |
| RCP8.5 | 2041–2070 | -12.55 | -9.79 | -10.55 | 16.67 |
| | 2071–2099 | -9.53 | -16.09 | -13.74 | 22.66 |

Generally, the interactions of precipitation, temperature, and evapotranspiration can be used to describe how climate change has affected the hydrology of a catchment. Reduced rainfall lowers surface runoff, while rising temperatures enhanced an increase in evapotranspiration that would have caused the runoff to decline. In this regard, the correlation between precipitation and the simulated surface runoff, groundwater and total water yield is positive, while the relationship between the temperature and hydrological process are positive only for PET. However, the relationship between the precipitation and temperature with the hydrological process varies with the RCPs and period of the scenario. Changes in precipitation, the expected decline in groundwater and total water yields, for instance, is larger under both RCPs for the period 2071-2100. However, under RCP4.5, the reduction in surface runoff is only more pronounced during the years 2071–2099 (-14.71%). Figure10 illustrates how the decline in surface water under RCP8.5 is greater throughout the period 2040–2070 (-12.55%). This demonstrates that changes in precipitation is not the only factor affecting surface water. This result was in line with previous studies on the Nile River. In the upper Nile, Coffel et al., (2019) found that hot, dry years are occurring more frequently due to rising regional temperatures.

The spatial distribution of the hydrological process under the projected climate change for RCP4.5 and RCP8.5 is presented in the following Figures. The catchment's surface runoff, groundwater, and water yield were all reported to have decreased due to climate change, but the yields in each sub-basin varied. While temperature changes are projected to have indirect effects, spatiotemporal patterns and magnitudes of precipitation are predicted to directly disrupt the hydrological process. In both scenarios throughout the study projections taken into consideration, the upstream of the catchment is often anticipated to have the greatest decline in surface runoff, groundwater, and water yield. Under the RCP4.5 and RCP8.5 scenarios, the projection for the

decline in surface runoff and water yield is greater in the intermediate than in the far future. In both scenarios, the southwest is where surface runoff is expected to decline at the slowest rate over the intermediate term and the far future, followed by the northeast. The southwest corner has a higher rate of groundwater and water yield decline. The highest rise in PET is shown by RCP8.5 around the outlet of the catchment in the mid future projection.

In both scenarios, it was observed that the catchment's surface runoff had decreased as a result of climate change. The upstream of the catchment is frequently expected to have the highest decline in surface runoff in both scenarios considered in the study forecasts. The projected decline in surface runoff is greater in the intermediate term than in the far future, according to the RCP4.5 and RCP8.5 scenarios. Surface runoff is anticipated to decrease at the slowest rate in both scenarios over the far future and the intermediate term in the southwest, followed by the northeast. Reduced surface runoff in the catchment are all potential outcomes that could affect the catchment's water resource availability and exacerbate water shortages (Figure 14).

According to the result, climate change has caused a decline in the catchment's groundwater flow. The upstream of the catchment is frequently expected to have the biggest groundwater decline in all scenarios that take into account study forecasts. Groundwater levels are declining faster in the southwest corner. Water stress downstream could be made worse by the potential for lower groundwater levels in the catchment, which would affect the availability of water resources in the catchment (Figure 15).

The upstream of the catchment is expected to have the highest decrease in water yield under both scenarios considered in the study estimates. The projected loss in water yield is larger in the intermediate than in the far future, according to the RCP4.5 and RCP8.5 scenarios. The rate of water yield decline is greater in the southwest corner. The potential for a decreased total water yield in the catchment could affect the availability of water resources in the catchment and worsen water stress downstream (Figure 16).

In general, the increase in temperature and decline in annual and seasonal precipitation in both scenarios could be the cause of the catchment's decreased average annual flow. The possibility of reduced surface water, groundwater, and total water yield in the catchment could have an impact on the availability of water resources in the catchment and exacerbate water stress downstream.

The acceleration of the increasing water abstractions could also contribute to the further decline in water resources besides the climate change pressure.

Reduced soil moisture, which is necessary for plant growth and groundwater storage, may result from the increase in PET brought on by rising temperatures and a decline in precipitation. This may indicate decreased water availability for agricultural production, which will become a chronic problem for the farmers in the catchment whose only source of income is agriculture. Additionally, a rise in temperature increased PET, which in turn raised the requirement for irrigation.

Similar findings were supported by research on climate change in Africa, namely the sub-Sahara, which showed a range of warming trends in the inland subtropics, a recurrence of high hot events, an increase in aridity, and changes in rainfall (Serdeczny et al 2017). The effects of climate change, according to Serdeczny et al (2017), have a considerable impact on the region's undernourishment, infectious diseases, vulnerability to rain-fed agriculture, and flash flooding catastrophes.

The results also support the findings of Mengistu et al. (2021), which indicated that the Nile river is predicted to decline between the years of 2040 and 2070 and again between 2070 and 2099 as a result of decreased precipitation and higher evaporation rate. In their study on the effects of climate change on the Tekeze river basin's water sources, Gebremeskel and Kebede (2018) also predicted that surface runoff would decrease by 13% and 14%. The decline of the expected surface runoff under both RCPs due to climate change was also confirmed in the study by Shiferaw et al. (2018) using the ensemble of five GCMs.

## 4. Conclusion and Management Options

This study analyzed the impacts of climate change on the hydrological processes of Gilgel Gibe catchment; the impact of possible future climate change predicted using 6 GCMs, and their ensemble mean were analyzed with a physically based distributed hydrologic model (SWAT). Six RCMs and their ensemble mean, under CORDEX-Africa, were applied for the climate change study. Except for HIRHAM5, all RCM simulations show declining precipitation for scenarios with high and medium-low emissions. Regarding temperature, all RCM projections depict rising temperatures with varied degrees of change. For the hydrological effect analyses of climate

changes, the ensemble mean of the RCMs was utilized to avoid the significant variability of the different RCMs in forecasting precipitation and temperature. The usage of ensemble RCMs was discovered to be a suitable technique to assess the uncertainties of individual RCMs, despite the fact that there are still uncertainties in climate prediction. Surface runoff, groundwater, and total water yield will all be reduced due to the rise in temperature and anticipated decrease in precipitation, however potential evapotranspiration will rise. In general, the Gilgel Gibe catchment's water resources are highly sensitive to climate change.

In addition, the catchment's seasonal and yearly variation in temperature and precipitation shows an increase in hot and dry years that would result in severe water scarcity. As a result, the catchment is extremely susceptible to the effects of climate change, which could include warming and crop failures due to extended dry seasons. Increased water security in the catchment is a result of the decline in runoff. According to the study analysis, the SWAT simulations adequately indicate the impact estimates of climate change in the Gilgel Gibe catchments. To increase our comprehension of the change in the present and future climate, urgent attention must be given to the region's low availability and poor quality of hydro-climatic data.

The findings of this study provided important information on the influences of how the watershed hydrological process in Gilgel Gibe catchment respond to the changes in climate. This might help to develop effective water resource management initiatives. If the degraded sloppy lands are rehabilitated, the ground recharge increase and the surface runoff, which washes the topsoil into the Gilgel Gibe I dam, is reduced. Furthermore, the Gilgel Gibe I hydroelectric dam in the catchment should be buffered with proper management strategies. In general, the result highlights the need for regional developments and cooperation to urge strong climate-resilient management strategies and to counteract the rapid climate changes in the catchment.

## References:


Abbaspour, K. C. (2015). SWAT calibration and uncertainty programs. A User Manual, 17-66.

Akalu, M. M. (2022). GIS-based land suitability assessment for surface irrigation: a case of Gilgel Gibe watershed, Jimma Zone, Ethiopia. *Arabian Journal of Geosciences*, *15*(5), 1-13.

Al- Ghussain, L. (2019). Global warming: Review on driving forces and mitigation. Environmental Progress & Sustainable Energy, 38(1), 13-21.

Aneseyee, A. B., Soromessa, T., Elias, E., Noszczyk, T., & Feyisa, G. L. (2022). Evaluation of Water Provision Ecosystem Services Associated with Land Use/Cover and Climate Variability in the Winike Watershed, Omo Gibe Basin of Ethiopia. Environmental management, 69(2), 367-383.

Arnold, J. G., Srinivasan, R., Muttiah, R. S., & Williams, J. R. (1998). Large area hydrologic modeling and assessment part I: model development 1. JAWRA Journal of the American Water Resources Association, 34(1), 73-89.

Ayele, G. T., Teshale, E. Z., Yu, B., Rutherfurd, I. D., & Jeong, J. (2017). Streamflow and sediment yield prediction for watershed prioritization in the Upper Blue Nile River Basin, Ethiopia. Water, 9(10), 782.

Behulu, F., Mutua, B. M., Gadissa, T., & Nyadawa, M. (2018). The effect of climate change on loss of lake volume: case of sedimentation in central rift valley basin, Ethiopia.

Behulu, F., Mutua, B. M., Gadissa, T., & Nyadawa, M. (2018). The effect of climate change on loss of lake volume: case of sedimentation in central rift valley basin, Ethiopia.

Beyene, T.; Lettenmaier, D.P.; Kabat, P. Hydrologic impacts of climate change on the Nile River Basin:

Bhaga, T. D., Dube, T., Shekede, M. D., & Shoko, C. (2020). Impacts of climate variability and drought on surface water resources in Sub-Saharan Africa using remote sensing: A review. Remote Sensing, 12(24), 4184.

Chen, J., Brissette, F. P., Zhang, X. J., Chen, H., Guo, S., & Zhao, Y. (2019). Bias correcting climate model multi-member ensembles to assess climate change impacts on hydrology. Climatic Change, 153(3), 361-377.

Coffel, E. D., Keith, B., Lesk, C., Horton, R. M., Bower, E., Lee, J., & Mankin, J. S. (2019). Future hot and dry years worsen Nile Basin water scarcity despite projected precipitation increases. Earth's Future, 7(8), 967-977.

CSA (Central Statistical Authority) (2008). The 2007 Population and Housing Census of Ethiopia. Statistical Report. Addis Ababa: CSA, Federal Democratic Republic of Ethiopia, Ethiopia.

Daba, M. H. (2018). Modelling the impacts of climate change on surface runoff in Finchaa sub-basin, Ethiopia. Journal of the Science of Food and Agriculture, 2, 14-29.

Daba, M. H., & You, S. (2020). Assessment of climate change impacts on river flow regimes in the upstream of Awash Basin, Ethiopia: based on IPCC fifth assessment report (AR5) climate change scenarios. Hydrology, 7(4), 98.

Dibaba, W. T., Miegel, K., & Demissie, T. A. (2019). Evaluation of the CORDEX regional climate models performance in simulating climate conditions of two catchments in Upper Blue Nile Basin. Dynamics of Atmospheres and Oceans, 87, 101104.

Duguma, B., & Janssens, G. P. (2021). Assessment of livestock feed resources and coping strategies with dry season feed scarcity in mixed crop–livestock farming systems around the gilgel gibe catchment, Southwest Ethiopia. Sustainability, 13(19), 10713.

Fanta, S. S., Namara, W. G., & Yesuf, M. B. (2022). Assessment of water supply and demand in Gilgel Gibe watershed, southwest Ethiopia. Sustainable Water Resources Management, 8(4), 1-28.

FAO/UNESCO. (1995). The digital soil map of the world, food and agriculture organization of the United Nations. Food and Agriculture Organization of the United Nations.

Fentaw, F., Hailu, D., Nigussie, A., & Melesse, A. M. (2018). Climate change impact on the hydrology of Tekeze Basin, Ethiopia: projection of rainfall-runoff for future water resources planning. Water Conservation Science and Engineering, 3(4), 267-278.

Gadissa, T., Nyadawa, M., Behulu, F., & Mutua, B. (2018). The effect of climate change on loss of lake volume: case of sedimentation in central rift valley basin, Ethiopia. Hydrology, 5(4), 67.

Gebre, S. L., Tadele, K., & Mariam, B. G. (2015). Potential impacts of climate change on the hydrology and water resources availability of Didessa Catchment, Blue Nile River Basin, Ethiopia. J. Geol. Geosci, 4, 193.

Gebremeskel, G., & Kebede, A. (2018). Estimating the effect of climate change on water resources: Integrated use of climate and hydrological models in the Werii watershed of the Tekeze river basin, Northern Ethiopia. Agriculture and Natural Resources, 52(2), 195-207.

Gedefaw, M., Wang, H., Yan, D., Qin, T., Wang, K., Girma, A., ... & Abiyu, A. (2019). Water resources allocation systems under irrigation expansion and climate change scenario in Awash River Basin of Ethiopia. Water, 11(10), 1966.
Geleta, C. D., & Gobosho, L. (2018). Climate change induced temperature prediction and bias correction in Finchaa watershed. Agric. Environ. Sci, 18, 324-337.
Getachew, B., Manjunatha, B. R., & Bhat, H. G. (2021). Modeling projected impacts of climate and land use/land cover changes on hydrological responses in the Lake Tana Basin, upper Blue Nile River Basin, Ethiopia. Journal of Hydrology, 595, 125974.
Global Facility for Disaster Reduction and Recovery (GFDRR), (2011). Climate Risk and Adaptation Country Profile: Ethiopia; World Bank: Washington, DC, USA.
Hasan, E., Tarhule, A., Kirstetter, P. E., Clark III, R., & Hong, Y. (2018). Runoff sensitivity to climate change in the Nile River Basin. Journal of hydrology, 561, 312-321.
Hassaballah, K., Mohamed, Y. A., & Uhlenbrook, S. (2019). The long-term trends in hydro-climatology of the Dinder and Rahad basins, Blue Nile, Ethiopia/Sudan. International Journal of Hydrology Science and Technology, 9(6), 690-712.
Herman, J. D., Quinn, J. D., Steinschneider, S., Giuliani, M., & Fletcher, S. (2020). Climate adaptation as a control problem: Review and perspectives on dynamic water resources planning under uncertainty. Water Resources Research, 56(2), e24389.
Hundera, H., Mpandeli, S., & Bantider, A. (2019). Smallholder farmers' awareness and perceptions of climate change in Adama district, central rift valley of Ethiopia. Weather and Climate Extremes, 26, 100230.
Implications of the 2007 IPCC scenarios. Clim. Chang. 2010, 100, 433–461.
Kasperson, J. X., Kasperson, R. E., Turner, B. L., Hsieh, W., & Schiller, A. (2022). Vulnerability to global environmental change. In The social contours of risk (pp. 245-285). Routledge.
Kephe, P. N., Ayisi, K. K., & Petja, B. M. (2021). Challenges and opportunities in crop simulation modelling under seasonal and projected climate change scenarios for crop production in South Africa. Agriculture & Food Security, 10(1), 1-24.
Lindersson, S., Brandimarte, L., Mård, J., & Di Baldassarre, G. (2020). A review of freely accessible global datasets for the study of floods, droughts and their interactions with human societies. Wiley Interdisciplinary Reviews: Water, 7(3), e1424.
Luo, M., Liu, T., Meng, F., Duan, Y., Frankl, A., Bao, A., & De Maeyer, P. (2018). Comparing bias correction methods used in downscaling precipitation and temperature from regional climate models: a case study from the Kaidu River Basin in Western China. Water, 10(8), 1046.
Madsen, H., Lawrence, D., Lang, M., Martinkova, M., & Kjeldsen, T. R. (2014). Review of trend analysis and climate change projections of extreme precipitation and floods in Europe. Journal of Hydrology, 519, 3634-3650.
Mengistu, D., Bewket, W., Dosio, A., & Panitz, H. J. (2021). Climate change impacts on water resources in the upper blue nile (Abay) river basin, ethiopia. Journal of Hydrology, 592, 125614.
Mengistu, D., Bewket, W., Dosio, A., & Panitz, H. J. (2021). Climate change impacts on water resources in the upper blue nile (Abay) river basin, ethiopia. Journal of Hydrology, 592, 125614.
Mengistu, D., Bewket, W., Dosio, A., & Panitz, H. J. (2021). Climate change impacts on water resources in the upper blue nile (Abay) river basin, ethiopia. Journal of Hydrology, 592, 125614.
Mohammed, M., Biazn, B., & Belete, M. D. (2020). Hydrological impacts of climate change in Tikur Wuha watershed, Ethiopian Rift Valley Basin. J Environ Earth Sci, 10(2), 28-49.
Moriasi, D. N., Arnold, J. G., Van Liew, M. W., Bingner, R. L., Harmel, R. D., & Veith, T. L. (2007). Model evaluation guidelines for systematic quantification of accuracy in watershed simulations. Transactions of the ASABE, 50(3), 885-900.
Muluneh, A., Bewket, W., Keesstra, S., & Stroosnijder, L. (2017). Searching for evidence of changes in extreme rainfall indices in the Central Rift Valley of Ethiopia. Theoretical and Applied Climatology, 128(3), 795-809.

Neitsch, S. L., Arnold, J. G., Kiniry, J. R., & Williams, J. R. (2011). Soil and water assessment tool theoretical documentation version 2009. Texas Water Resources Institute.

Payne, A. E., Demory, M. E., Leung, L. R., Ramos, A. M., Shields, C. A., Rutz, J. J., ... & Ralph, F. M. (2020). Responses and impacts of atmospheric rivers to climate change. Nature Reviews Earth & Environment, 1(3), 143-157.

Pérez, D. M. G., Martín, J. M. M., Martínez, J. M. G., & Sáez-Fernández, F. J. (2020). An analysis of the cost of water supply linked to the tourism industry. An application to the case of the Island of Ibiza in Spain. Water, 12(7), 2006.

Radecki-Pawlik, A., Wałęga, A., Młyński, D., Młocek, W., Kokoszka, R., Tokarczyk, T., & Szalińska, W. (2020). Seasonality of mean flows as a potential tool for the assessment of ecological processes: Mountain rivers, Polish Carpathians. Science of the Total Environment, 716, 136988.

Rathjens, H.; Bieger, K.; Srinivasan, R.; Chaubey, I.; Arnold, J.G. CMhyd User Manual. 2016. Available online: http://swat.tamu.edu/software/cmhyd/ (accessed on 2 January 2020).

Ribeiro, K., Pacheco, F. S., Ferreira, J. W., de Sousa‐ Neto, E. R., Hastie, A., Krieger Filho, G. C., ... & Ometto, J. P. (2021). Tropical peatlands and their contribution to the global carbon cycle and climate change. Global Change Biology, 27(3), 489-505.

Rickards, N., Thomas, T., Kaelin, A., Houghton-Carr, H., Jain, S. K., Mishra, P. K., ... & Rees, G. (2020). Understanding future water challenges in a highly regulated Indian river basin—modelling the impact of climate change on the hydrology of the Upper Narmada. Water, 12(6), 1762.

Roy, T., Valdés, J. B., Lyon, B., Demaria, E. M., Serrat-Capdevila, A., Gupta, H. V., ... & Durcik, M. (2018). Assessing hydrological impacts of short-term climate change in the Mara River basin of East Africa. Journal of Hydrology, 566, 818-829.

Serdeczny, O., Adams, S., Baarsch, F., Coumou, D., Robinson, A., Hare, W., ... & Reinhardt, J. (2017). Climate change impacts in Sub-Saharan Africa: from physical changes to their social repercussions. Regional Environmental Change, 17(6), 1585-1600.

Shahvari, N., Khalilian, S., Mosavi, S. H., & Mortazavi, S. A. (2019). Assessing climate change impacts on water resources and crop yield: a case study of Varamin plain basin, Iran. Environmental monitoring and assessment, 191(3), 1-12.

Shao, Y., Jiang, Q. O., Wang, C., Wang, M., Xiao, L., & Qi, Y. (2020). Analysis of critical land degradation and development processes and their driving mechanism in the Heihe River Basin. Science of the Total Environment, 716, 137082.

Sheffield, J., Wood, E. F., Pan, M., Beck, H., Coccia, G., Serrat‐ Capdevila, A., & Verbist, K. (2018). Satellite remote sensing for water resources management: Potential for supporting sustainable development in data‐ poor regions. Water Resources Research, 54(12), 9724-9758.

Shiferaw, H., Gebremedhin, A., Gebretsadkan, T., & Zenebe, A. (2018). Modelling hydrological response under climate change scenarios using SWAT model: the case of Ilala watershed, Northern Ethiopia. Modeling Earth Systems and Environment, 4(1), 437-449.

Shiferaw, H., Gebremedhin, A., Gebretsadkan, T., & Zenebe, A. (2018). Modelling hydrological response under climate change scenarios using SWAT model: the case of Ilala watershed, Northern Ethiopia. Modeling Earth Systems and Environment, 4(1), 437-449.

Shiru, M. S., Shahid, S., Dewan, A., Chung, E. S., Alias, N., Ahmed, K., & Hassan, Q. K. (2020). Projection of meteorological droughts in Nigeria during growing seasons under climate change scenarios. Scientific reports, 10(1), 1-18.

Shu, J., Qu, J. J., Motha, R., Xu, J. C., & Dong, D. F. (2018, July). Impacts of climate change on hydropower development and sustainability: a review. In IOP Conference Series: Earth and Environmental Science (Vol. 163, No. 1, p. 012126). IOP Publishing.

Tesfahunegn, G. B., & Gebru, T. A. (2020). Smallholder farmers' level of understanding on the impacts of climate change on water resources in northern Ethiopia catchment. GeoJournal, 1-19.

Teutschbein, C., & Seibert, J. (2012). Bias correction of regional climate model simulations for hydrological climate-change impact studies: Review and evaluation of different methods. Journal of hydrology, 456, 12-29.
Teutschbein, C., & Seibert, J. (2012). Bias correction of regional climate model simulations for hydrological climate-change impact studies: Review and evaluation of different methods. Journal of hydrology, 456, 12-29.
Van Velthuizen, H., & Verelst, L. (2009). Harmonized World Soil Database Version 1.1.

Wedajo, A. G., Fufa, F., & Mentsiro, A. A. (2022). Assessment of Spatial Soil Erosion Using RUSLE Model Integration with GIS and RS Tools: The Case of Gilgel Gibe-I Catchment, South West Ethiopia. American Journal of Bioscience and Bioengineering, 10(1), 10-22.

Woldeab, B., Ambelu, A., Mereta, S. T., & Beyene, A. (2019). Effect of watershed land use on tributaries' water quality in the east African Highland. Environmental monitoring and assessment, 191(1), 1-13.

Worku, G., Teferi, E., Bantider, A., & Dile, Y. T. (2020). Statistical bias correction of regional climate model simulations for climate change projection in the Jemma sub-basin, upper Blue Nile Basin of Ethiopia. Theoretical and Applied Climatology, 139(3), 1569-1588.

Worku, G., Teferi, E., Bantider, A., & Dile, Y. T. (2021). Modelling hydrological processes under climate change scenarios in the Jemma sub-basin of upper Blue Nile Basin, Ethiopia. Climate Risk Management, 31, 100272.

Yang, D., Yang, Y., & Xia, J. (2021). Hydrological cycle and water resources in a changing world: A review. Geography and Sustainability, 2(2), 115-122.

Zhang, B., Shrestha, N. K., Daggupati, P., Rudra, R., Shukla, R., Kaur, B., & Hou, J. (2018). Quantifying the impacts of climate change on streamflow dynamics of two major rivers of the Northern Lake Erie Basin in Canada. Sustainability, 10(8), 2897.

Figure

Click here to access/download;Figure;Figure 1 Location and elevation map of study area.docx

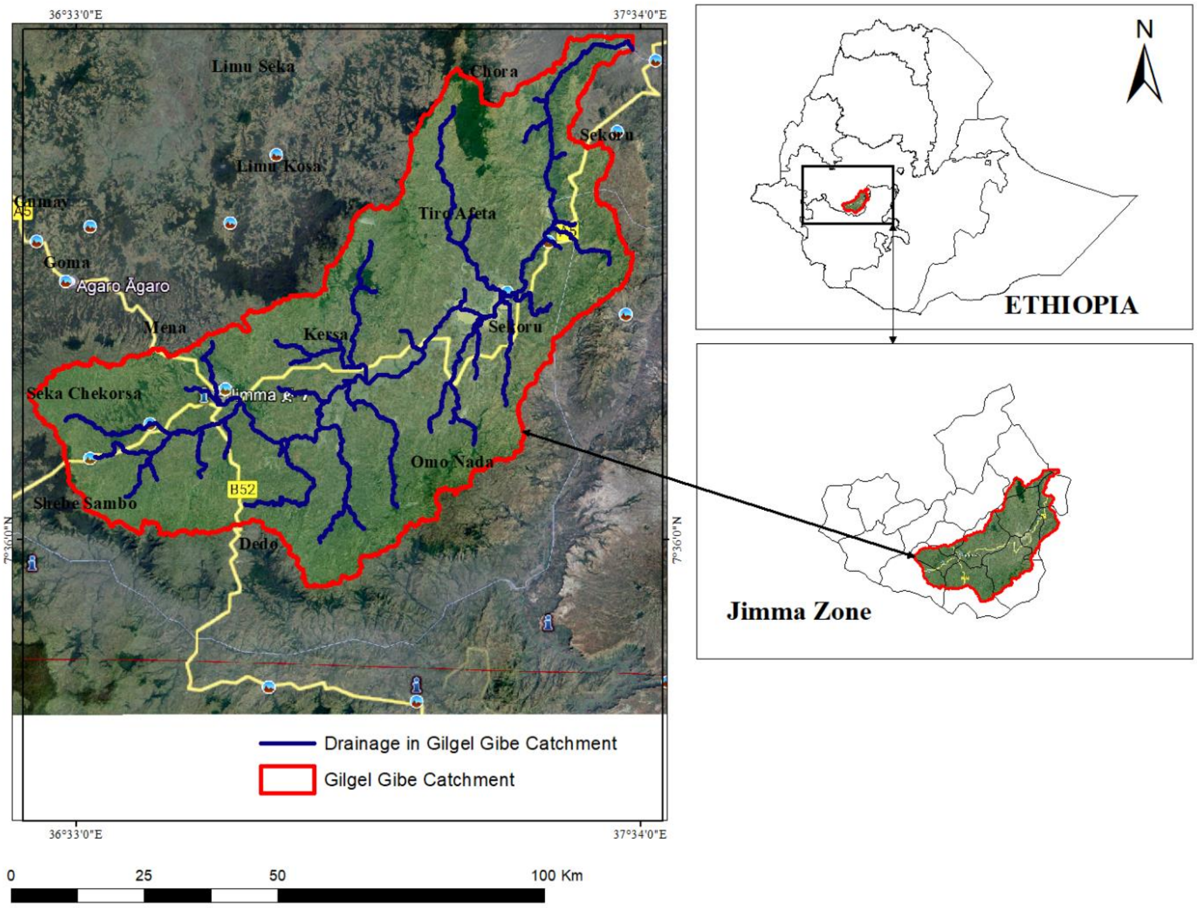


Figure 1 Location and elevation map of study area

Click here to access/download;Figure;Figure 2 The dominant soils of Gilgel Gibe catchment.docx

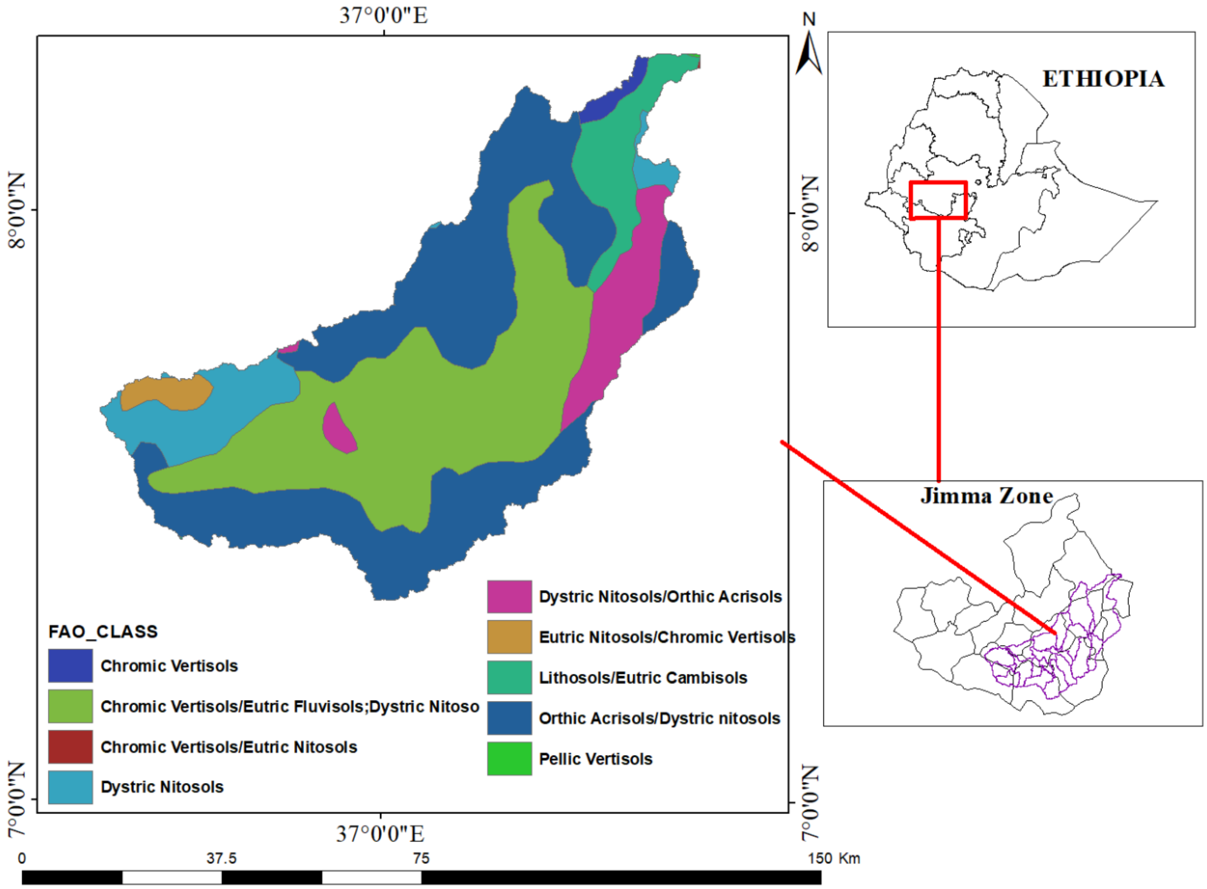


Figure 1 The dominant soils of Gilgel Gibe catchment

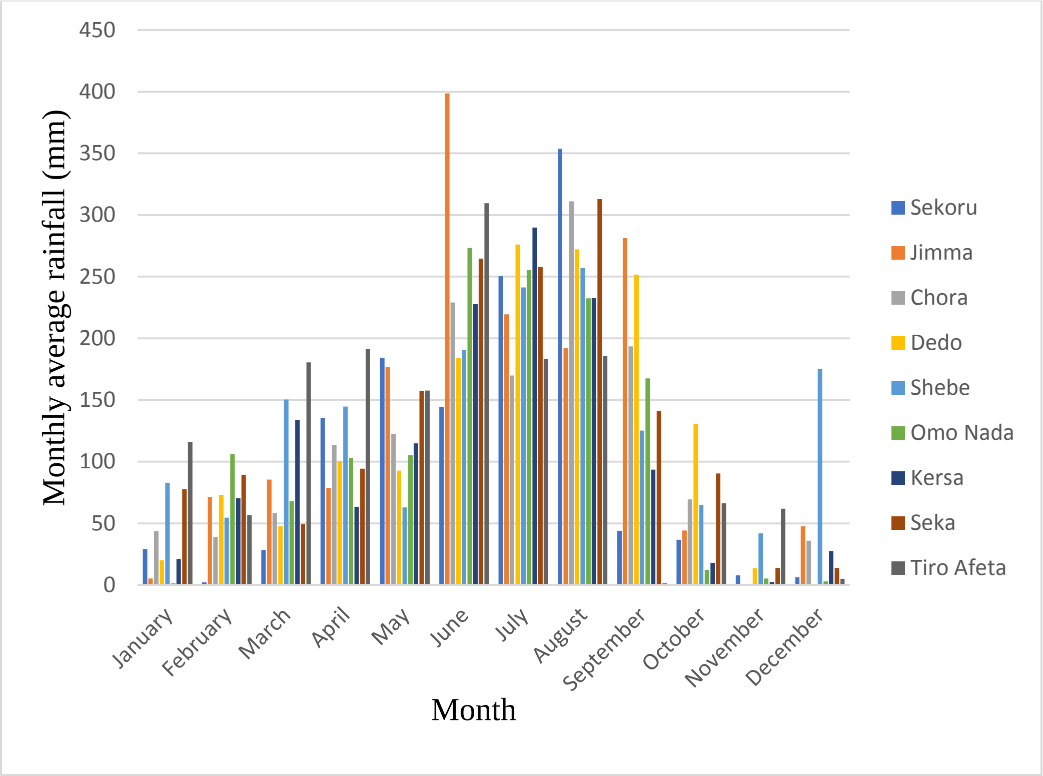


Figure 1 Average monthly rainfall of the selected stations

Figure

Click here to access/download;Figure;Figure 4 Monthly maximum and minimum te.docx

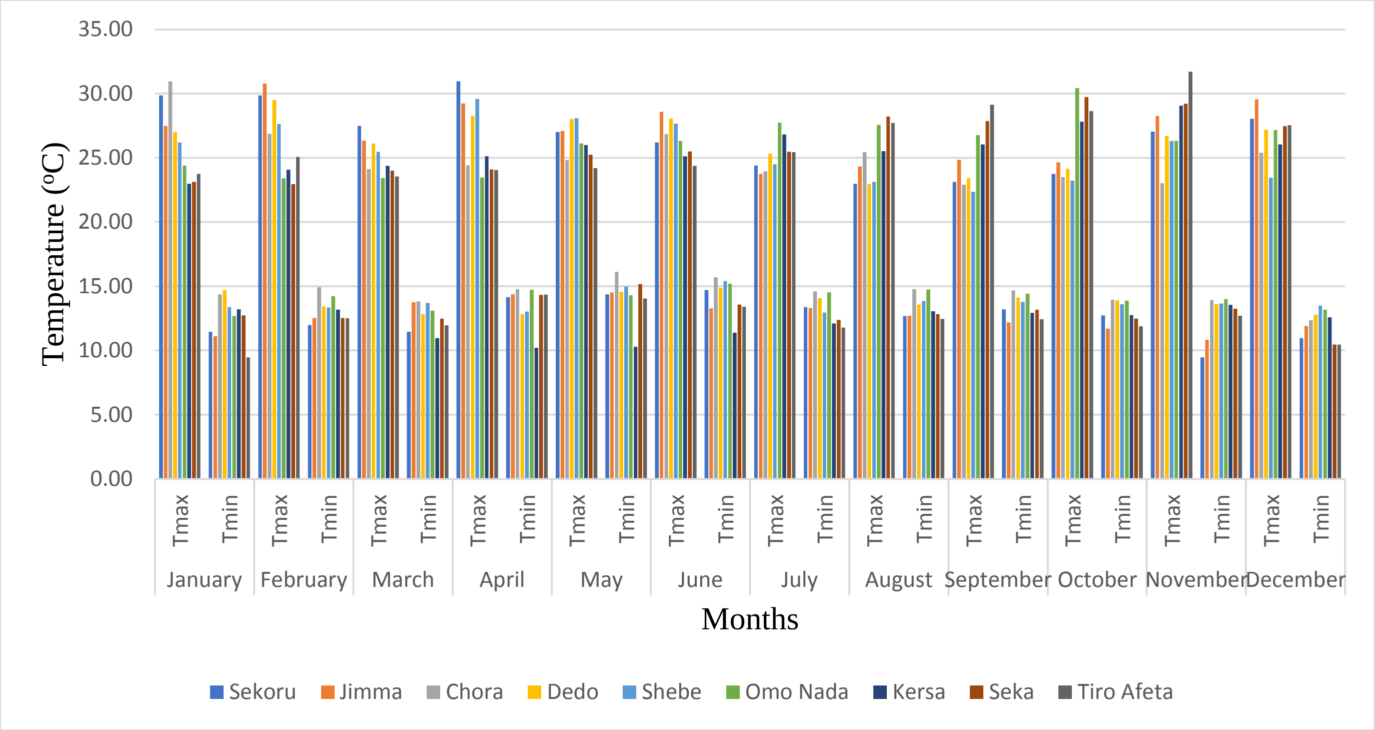


Figure 1 Monthly maximum and minimum temperature of the selected stations

Click here to access/download;Figure;Figure 5 Flowchart of ArcSWAT processing steps.docx

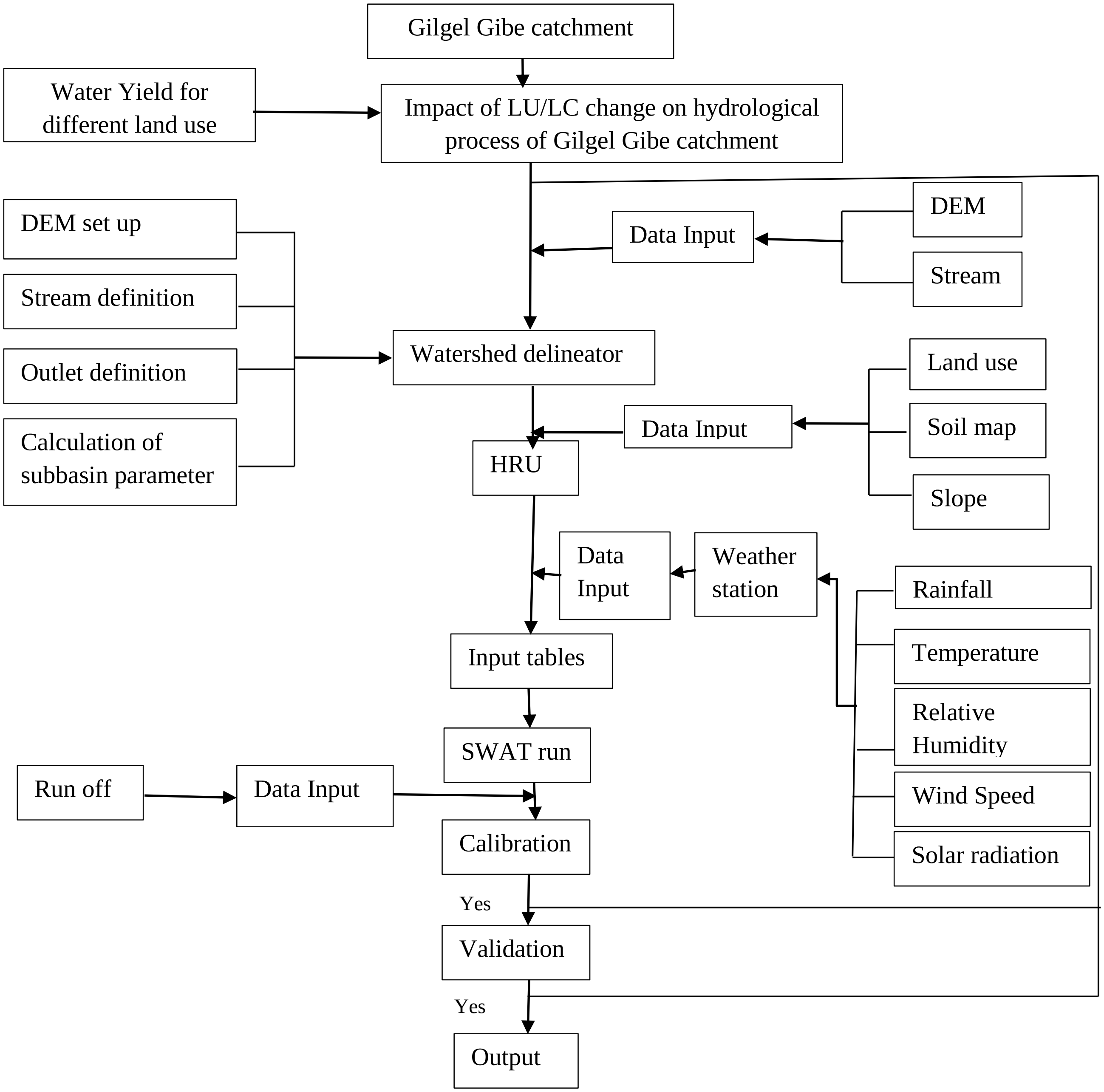


Figure 1 Flowchart of ArcSWAT processing steps for the Gilgel Gibe catchment

Click here to access/download;Figure;Figure 6 Sub-watersheds of the Gilgel Gibe Catchment.docx

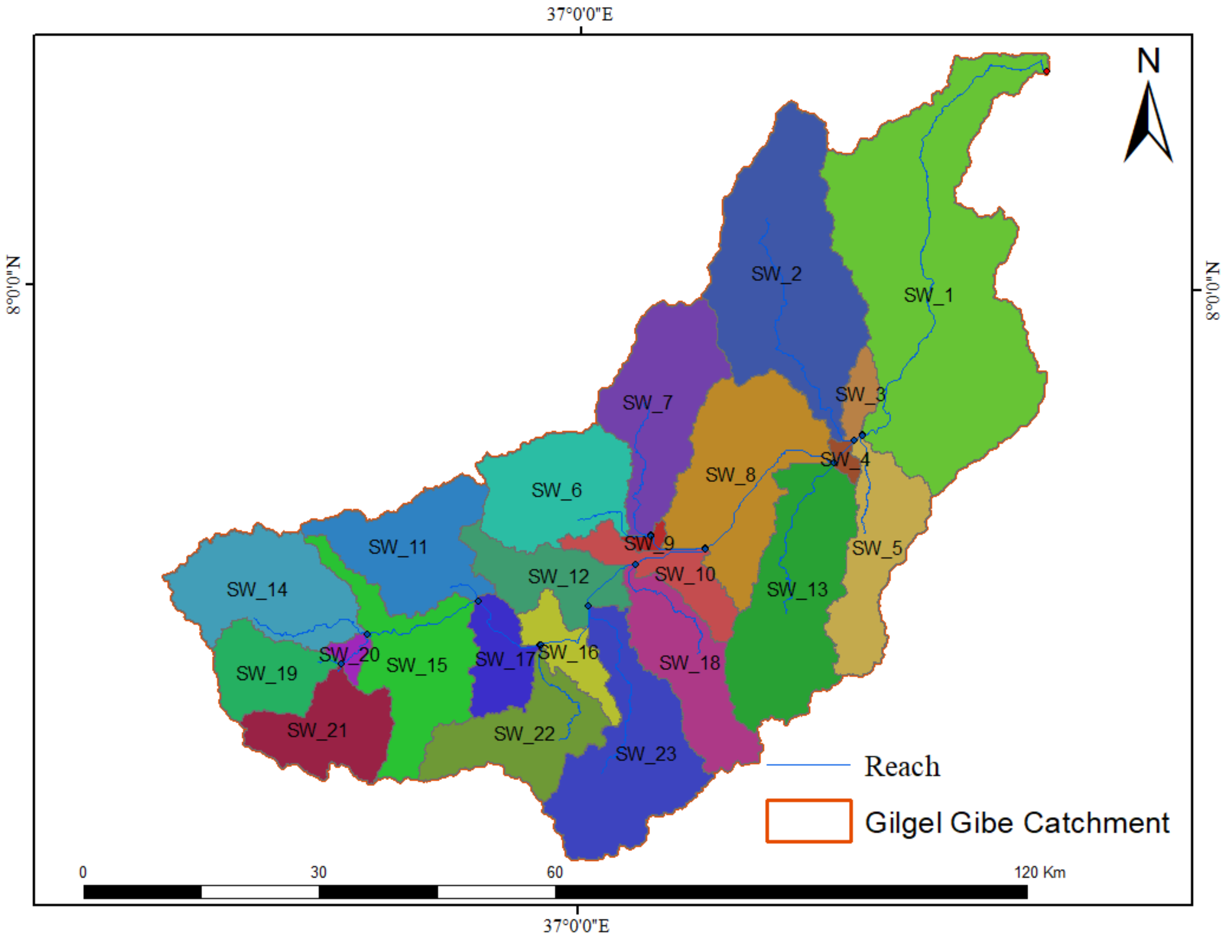


Figure 1 Sub-watersheds of the Gilgel Gibe Catchment

Figure Click here to access/download;Figure;Figure 7 Precipitation and temperature recording sta.docx

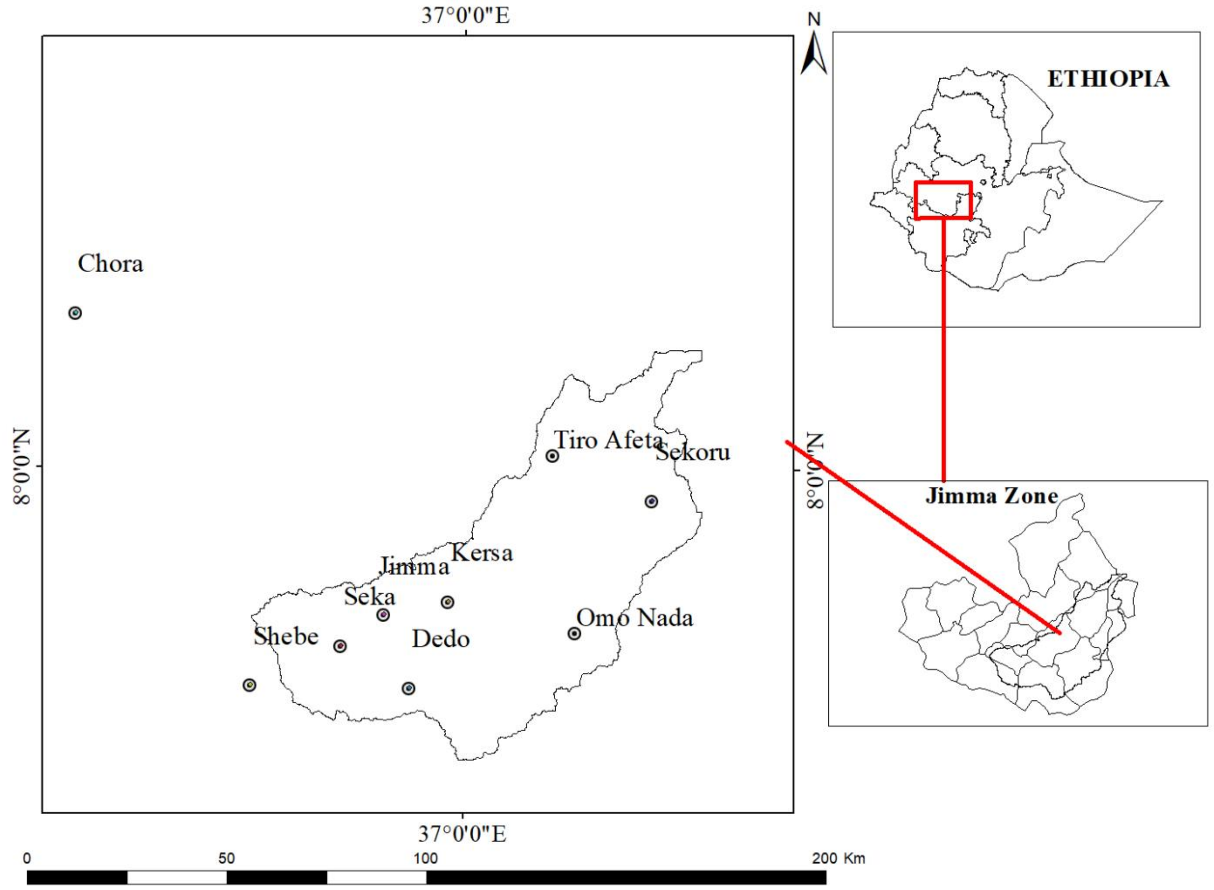


Figure 1 Precipitation and temperature recording stations

 Click here to access/download;Figure;Figure 8 Drainage in Gilgel Gibe catchment.docx

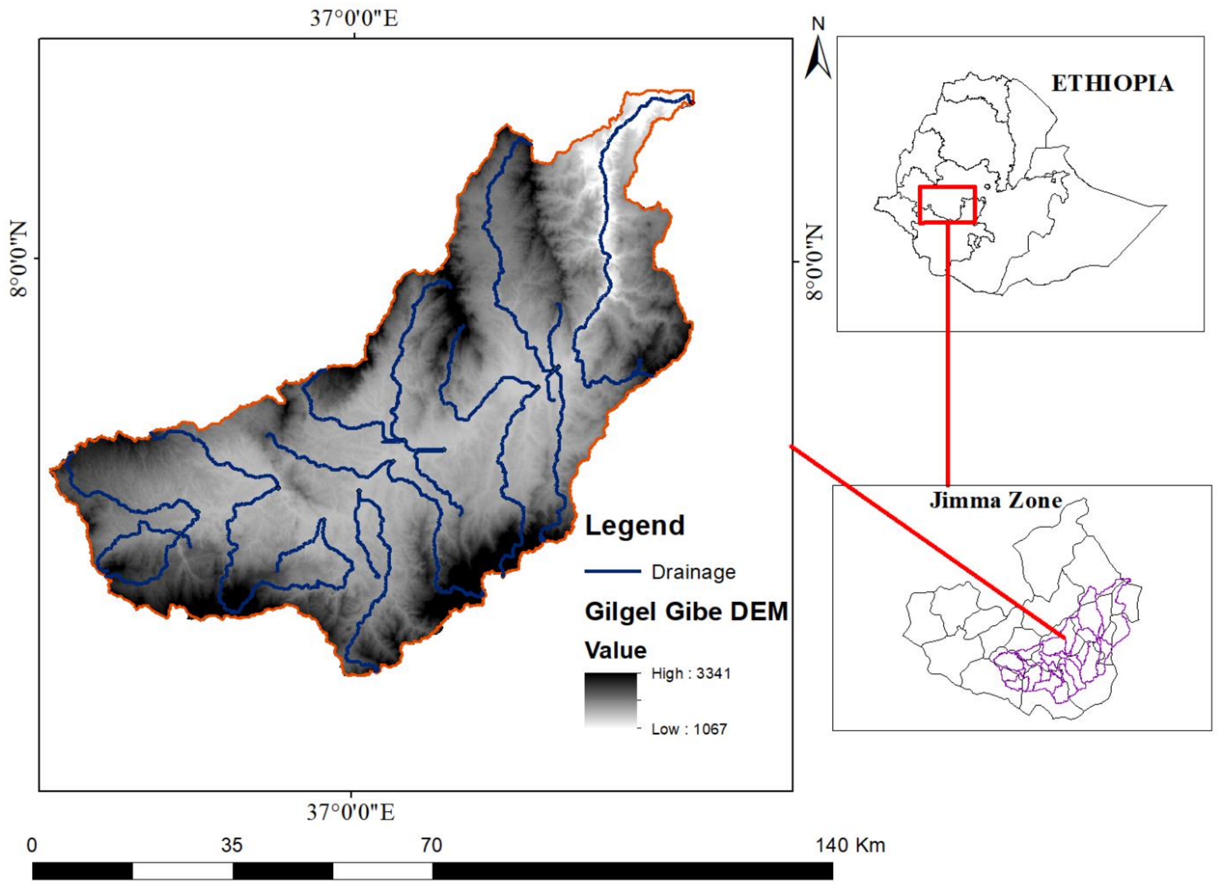


Figure 1 Drainage in Gilgel Gibe catchment

Click here to access/download;Figure;Figure 9 Calibration and validation of average monthly str.docx

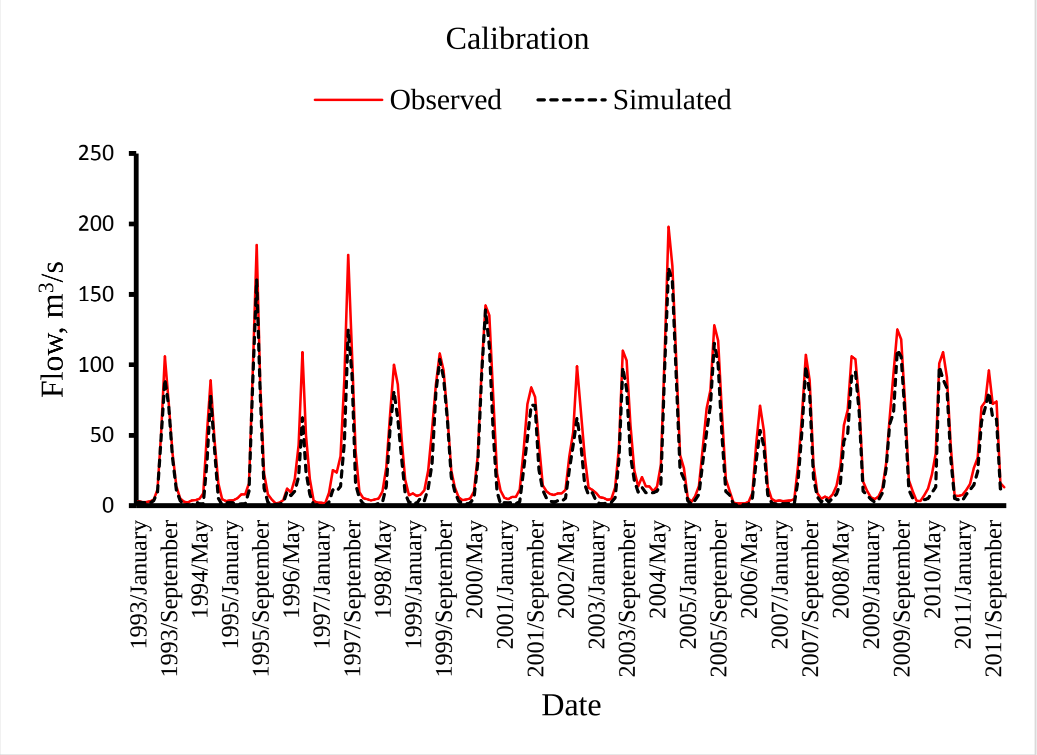


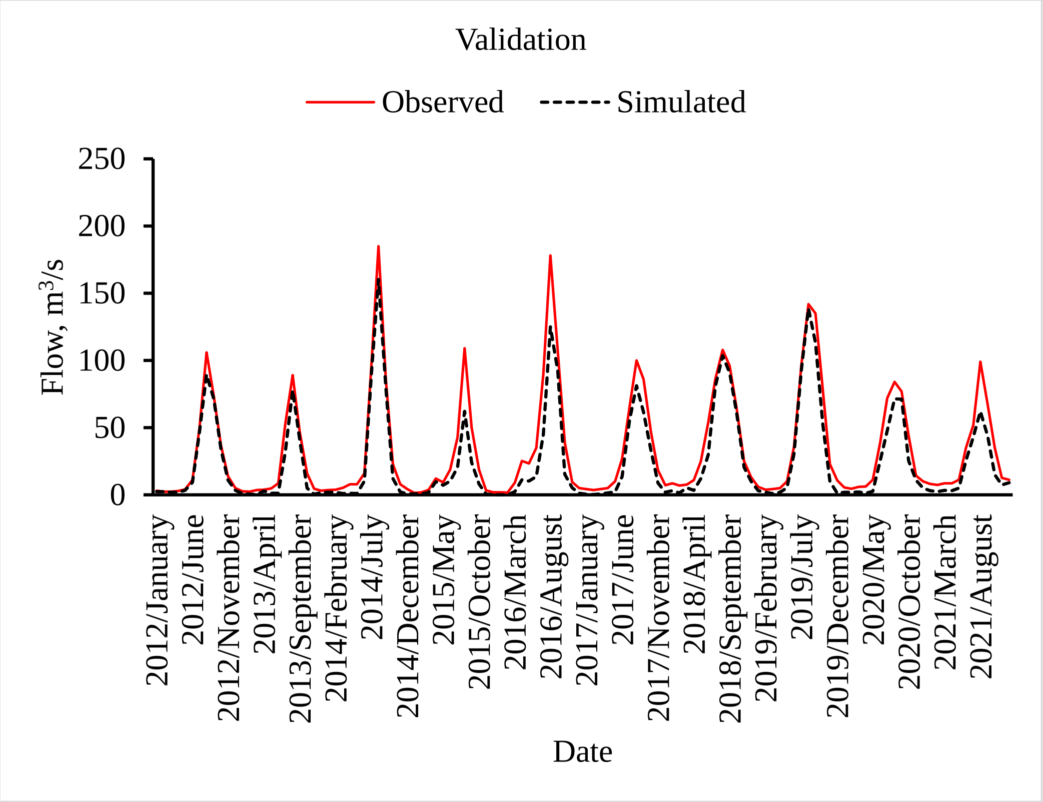


Figure 1 Calibration and validation of average monthly streamflow

Figure

Click here to access/download;Figure;Figure 10 Percentage change of projected average prec.docx

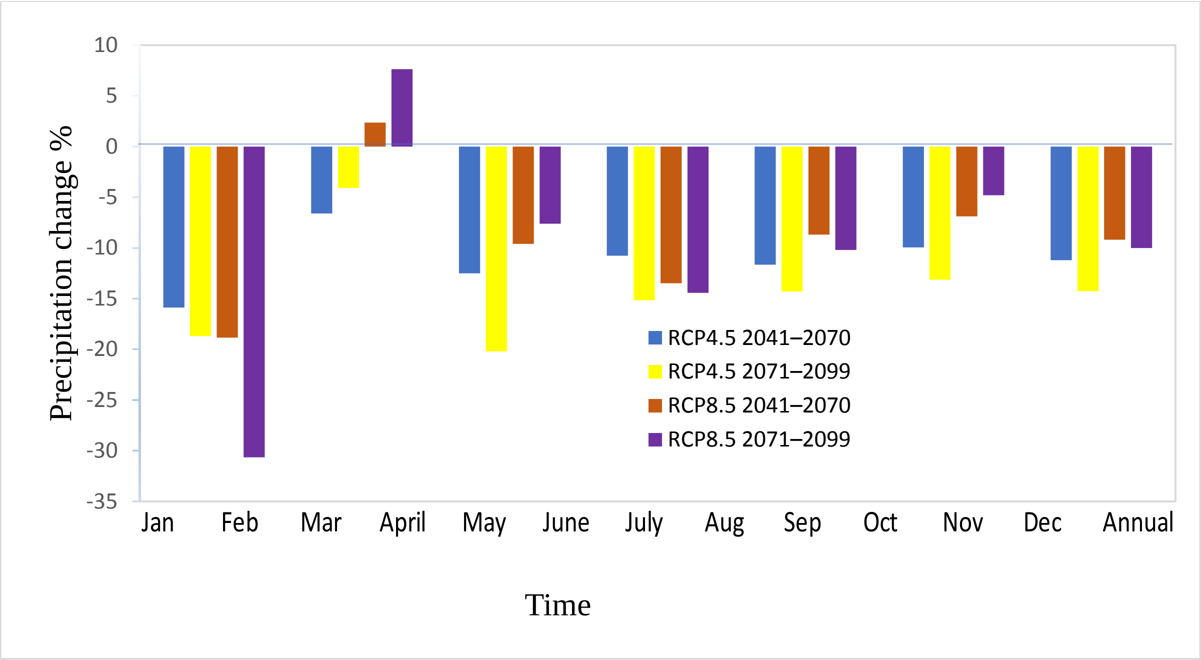


Figure 1 Percentage change of projected average precipitation in Gilgel Gibe catchment for 2041–2070 and 2071–2099 under RCP4.5 and RCP8.5

Click here to access/download;Figure;Figure 11 Seasonal projected change in mean maximum.docx

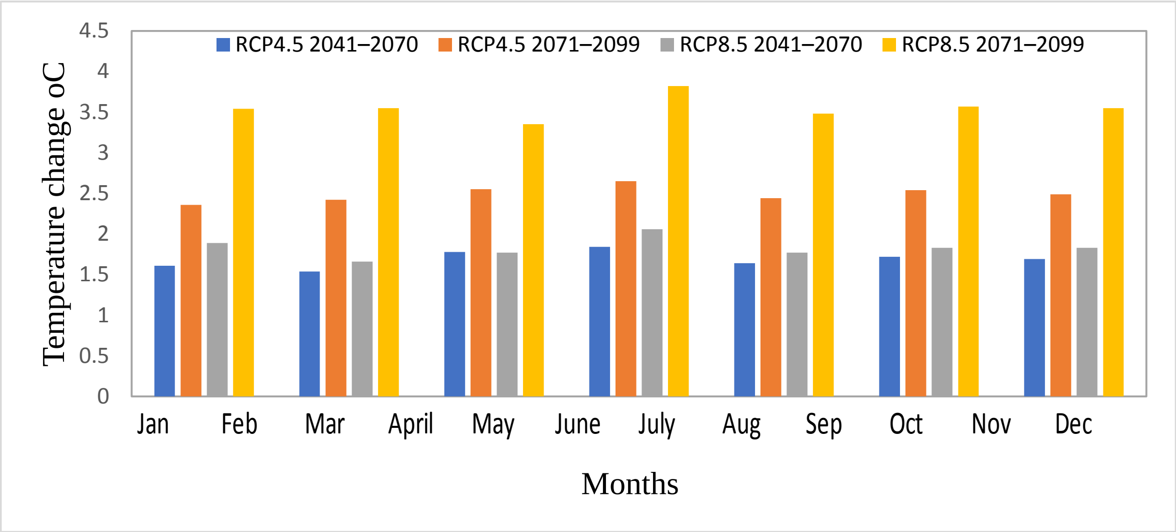


Figure 1 Seasonal projected change in mean maximum temperature in Gilgel gibe catchment for 2041–2070 and 2071–2099 under RCP4.5 and RCP8.5

Figure

Click here to access/download;Figure;Figure 12 Seasonal projected change in mean minimum te.docx

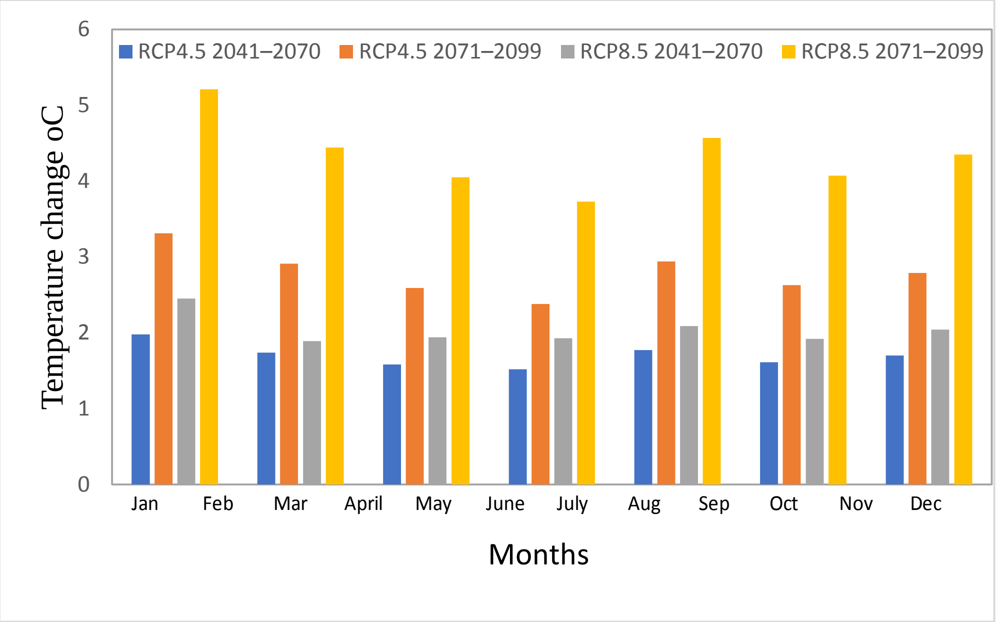


Figure 1 Seasonal projected change in mean minimum temperature in Gilgel gibe catchment for 2041–2070 and 2071–2099 under RCP4.5 and RCP8.5

Click here to access/download;Figure;Figure 13 Impacts of climate change on water balance.docx

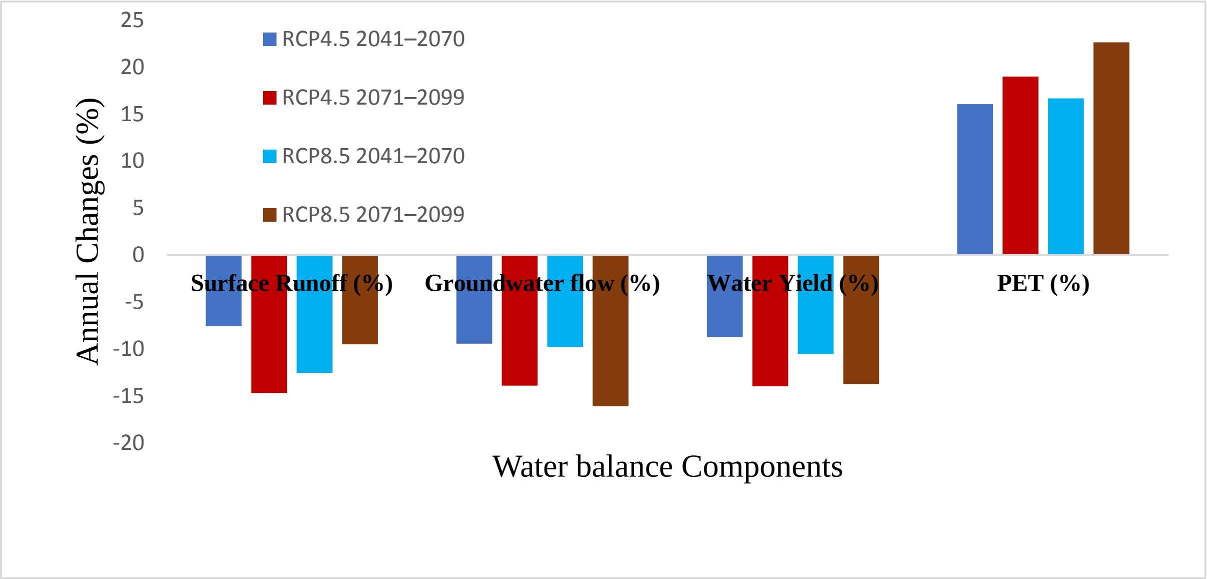


Figure 1 Impacts of climate change on water balance

Figure

Click here to access/download;Figure;Figure 14 Spatial patterns of the changes in surfaceR.docx

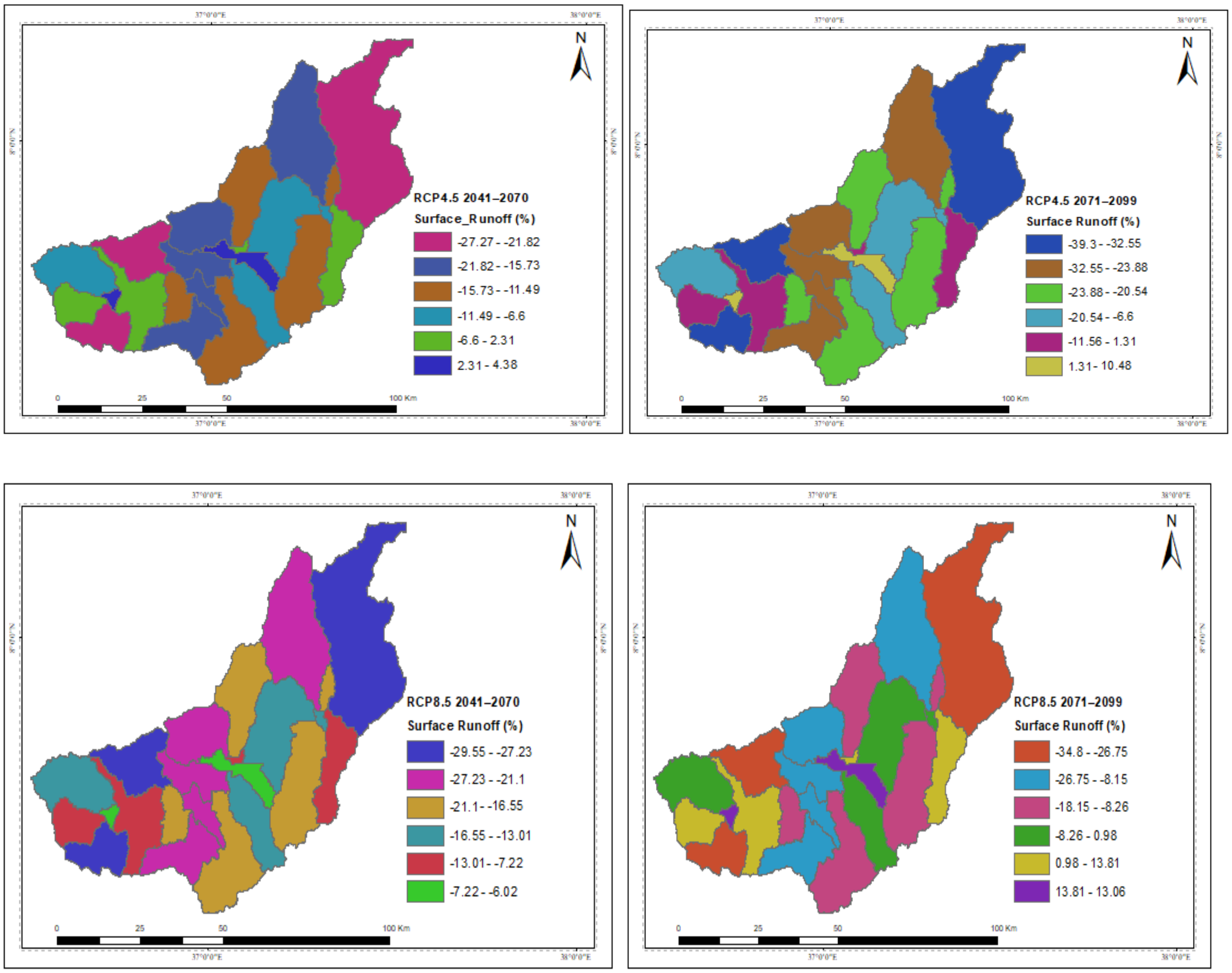


Figure 1 Spatial patterns of the changes in surface runoff due to climate change

Figure

Click here to access/download;Figure;Figure 15 Spatial patterns of the changes in groundwate.docx

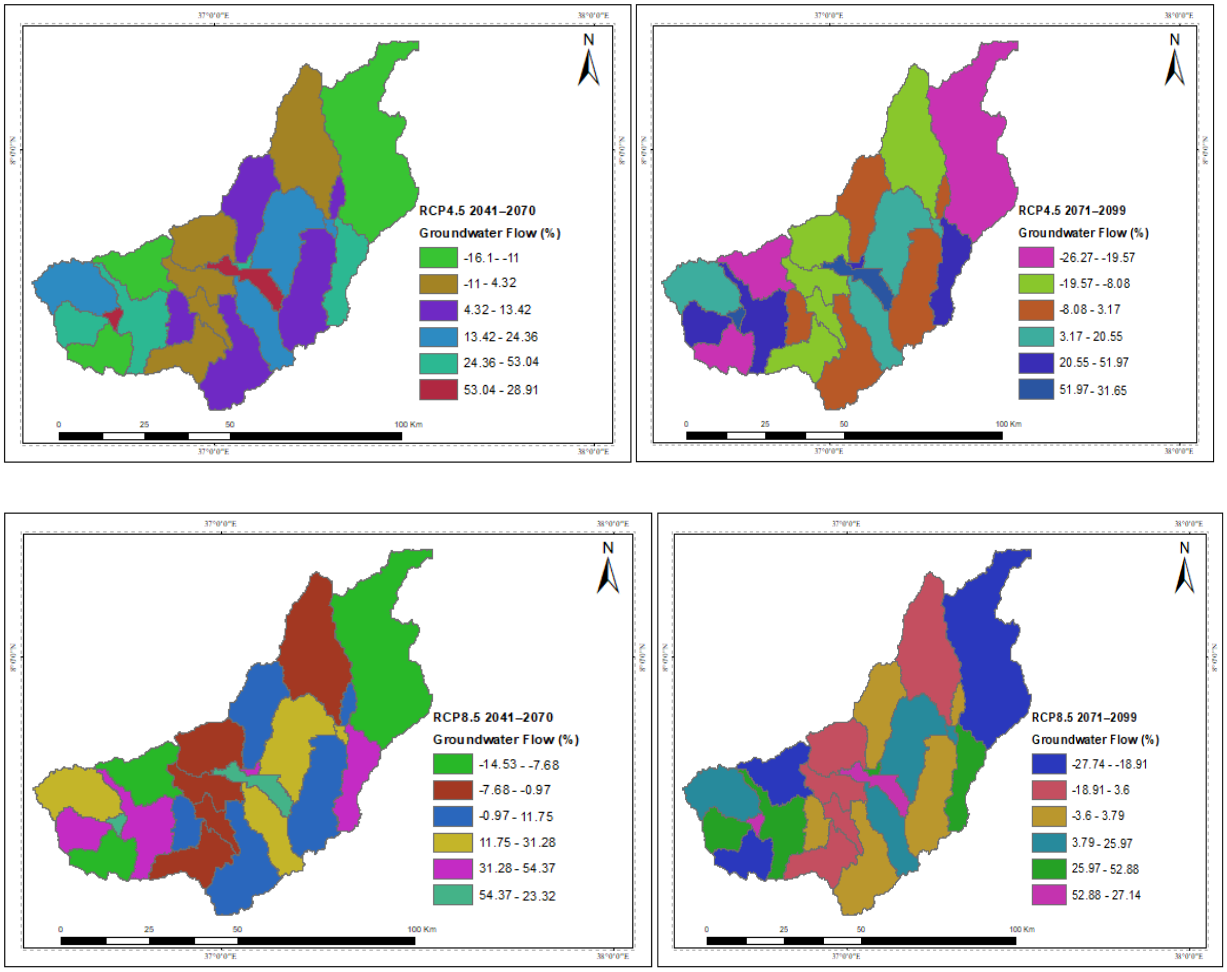


Figure 1 Spatial patterns of the changes in groundwater due to climate change

Figure

Click here to access/download;Figure;Figure 16 Spatial patterns of the changes in water yield.docx

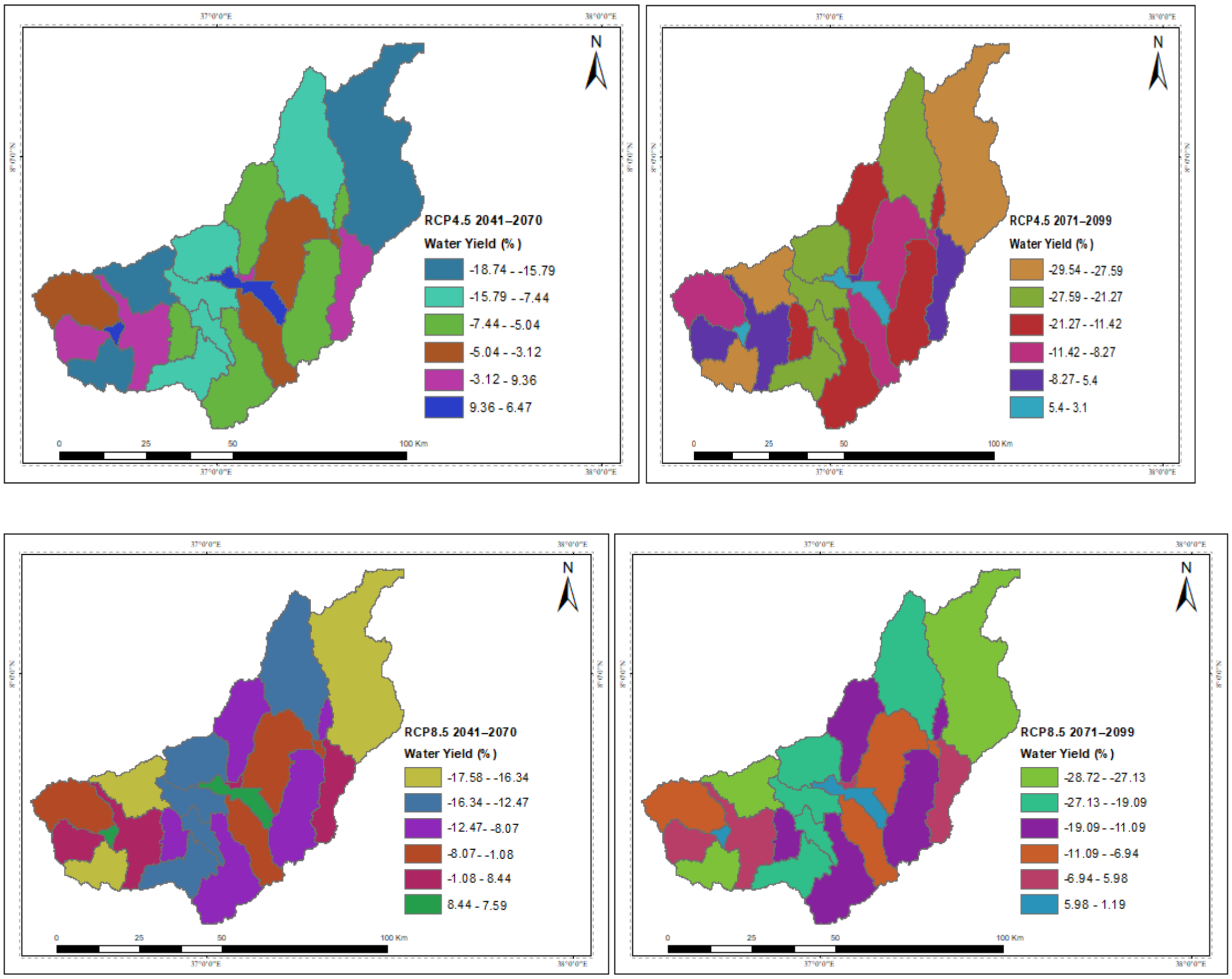


Figure 1 Spatial patterns of the changes in water yield due to climate change

Figure

Click here to access/download;Figure;Figure 17 Spatial patterns of the changes in PET.docx

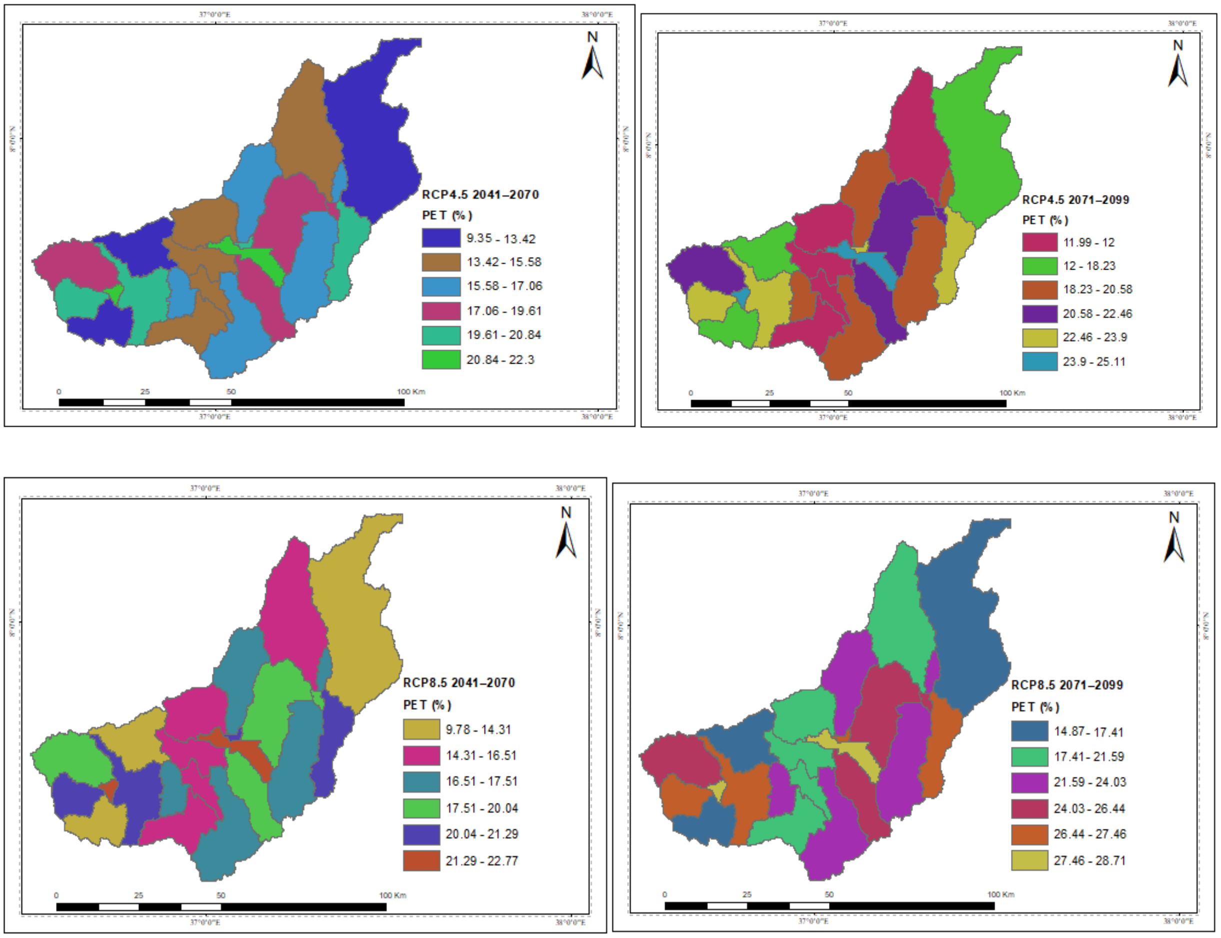


Figure 1 Spatial patterns of the changes in Potential Evapotranspiration (PET) due to climate change

Click here to access/download
**Supporting Information**
Conflict of Interest.docx

Click here to access/download
**Supporting Information**
CRediT author statement1.doc
