# Supplementary material for: The impacts of climate change on hydrological processes of Gilgel Gibe catchment, southwest Ethiopia
Source: PLoS One. 2023 Jun 28;18(6):e0287314. doi: 10.1371/journal.pone.0287314 (PMC10306186; doi:10.1371/journal.pone.0287314)
Supplement: Attachment — Submitted filename: Comments for authors.docx [file pone.0287314.s001.docx]

The work, " The Impacts of Climate Change on Hydrological Processes of Gilgel Gibe Catchment, Southwest Ethiopia" assesses the changes in hydrological parameters due to changing climate in the region. Overall the work is good. However, more exhaustive conclusion is further required to relate the changing climate impacts on hydrological processes and other forcing factors operating in the region.

**Some of the specific comments are listed below:**

Line 40 rephrase the sentence or rewrite the whole first paragraph. Authors had loosely written introduction. The main function of an introduction section is to contextualise your study, here it is somewhat missing.

Line 87, rephrase the beginning sentence of paragraph?

Line 109-114, Authors need to check last paragraph of introduction, please formulate explicitly your research questions and the novelty of your work and state clearly the specific paper objectives. Authors are requested go through results and conclusion thoroughly first and then write last paragraph of introduction.

Line 179-180, Authors have chosen Distribution mapping (DM) method for bias correction for both precipitation and temperature based on absolute error ranking. However, authors had discussed the studies where DM method have been used. It is suggested that the authors could have provided table in which absolute error value for other methods (Linear scaling (multiplicative), delta-change correction (multiplicative), precipitation local intensity scaling, and power transformation of precipitation with respect to DM) for Precipitation data and (Linear scaling (additive), delta-change correction (additive), variance scaling of temperature with respect to DM) for temperature data are provided. At least provide 2 methods for comparison.

Line 185-186, rephrase the sentence “Based on mean absolute error ranking, distribution mapping was ranked both, for temperature and precipitation corrections”.

Line 241-247, Landsat Thematic Mapper of 1991, Enhanced Thematic Mapper Plus of 2006, and Operational Land Imager or Thermal Infrared Sensor of 2021 were used for LULC classifications. Which LULC map is used for SWAT Model? Authors had employed different satellite data but didn’t mention LULC map used in the study. Please make it clear.

Line 257, Authors need to mention metrological station names only in place of their coordinates or put a table for name and coordinates of Met. Stations.

Line 266, Authors are advised either write all weather data used in the SWAT for simulation with warmup periods, calibration and validation or remove this sentence. Moreover, weather data from 1991 and 1992 were used as the model initialization phase (warm up) and not included in the final analysis.

Line 268, Same comment as of 257

Authors are advised check this paragraph “Sensitivity Analysis, Calibration and Validation”, it is misleading. Authors have performed analysis on daily observed flow or mean monthly flow, please make it clear.

Line 320-321, Sensitivity analysis for the simulated streamflow was performed using a daily observed flow to identify the most sensitive parameter with strong influence on model outputs.

Line 334-335	The calibration results on mean monthly flow show that SWAT model is able to capture the observed streamflow with R2, NSE, PBIAS and RSR of 0.76, 0.75, 1.02, and 0.49 respectively.

Line 530, Conclusion is not conclusive, make it clear particularly last two paragraphs of conclusion.

Figures, give appropriate number to figures, both in text and at the end.

Authors are suggested to modify the flow chat, there are many horrible mistakes in it.
