# Supplementary material for: The impacts of climate change on hydrological processes of Gilgel Gibe catchment, southwest Ethiopia
Source: PLoS One. 2023 Jun 28;18(6):e0287314. doi: 10.1371/journal.pone.0287314 (PMC10306186; doi:10.1371/journal.pone.0287314)
Supplement: Attachment — Submitted filename: Commets on PONE-D-22-23861_R1.docx [file pone.0287314.s004.docx]

Comment 15:

Flowchart needs to be changed, at least group the input files why authors are using detailed description about the SWAT inputs files. It’s better to be concise in flowchart. However, in order to show authors had worked rigorously doesn’t mean to provide details of every input file used.

Please check the figure below and figure 3.1 of manuscript “**Soil and Water Assessment Tool (SWAT) for Simulating the Sediment and Water Yield of Alpine Catchments – A Brief Review”**. Authors may get idea, how to group all the inputs and output layers.
