# Supplementary material for: The impacts of climate change on hydrological processes of Gilgel Gibe catchment, southwest Ethiopia
Source: PLoS One. 2023 Jun 28;18(6):e0287314. doi: 10.1371/journal.pone.0287314 (PMC10306186; doi:10.1371/journal.pone.0287314)
Supplement: Attachment — Submitted filename: Response to Reviewers.docx [file pone.0287314.s005.docx]

Thank you for the opportunity to submit a revised draft of our manuscript titled “The Impacts of Climate Change on Hydrological Processes of Gilgel Gibe Catchment, Southwest Ethiopia” for consideration for publication in the Journal of PLOS ONE. We sincerely appreciate the time and effort you and the reviewers have invested in providing feedback on our work, and we are grateful for the insightful comments and valuable improvements that have been made to our paper.

We have taken into consideration the majority of the suggestions and criticisms raised by the reviewers and have made appropriate revisions to the manuscript. The changes we have made in response to the reviewers’ comments are clearly indicated within the revised manuscript. For your convenience, we have also included a point-by-point response to the reviewers’ concerns in blue text below.

We hope that the revised manuscript meets the expectations of the Journal's editorial board and reviewers, and we believe that the final version of our work will provide a valuable contribution to the field of climate science. Please note that all page numbers referenced in our response correspond to the revised manuscript file with tracked changes.

Once again, we express our gratitude for the opportunity to revise and resubmit our manuscript for your consideration.

**Reviewer#1**
**Comment#1**: All comments have been addressed

**Response#1:** Thank you for taking the time to review our work. We would like to express our gratitude for your valuable feedback.

**Comment 15:** Flowchart needs to be changed, at least group the input files why authors are using detailed description about the SWAT inputs files. It’s better to be concise in flowchart. However, in order to show authors had worked rigorously doesn’t mean to provide details of every input file used.
Please check the figure below and figure 3.1 of manuscript “Soil and Water Assessment Tool (SWAT) for Simulating the Sediment and Water Yield of Alpine Catchments – A Brief Review”. Authors may get idea, how to group all the inputs and output layers.

**Comment 15:** Flowchart needs to be changed, at least group the input files why authors are using detailed description about the SWAT inputs files. It’s better to be concise in flowchart. However, in order to show authors had worked rigorously doesn’t mean to provide details of every input file used. Please check the figure below and figure 3.1 of manuscript “**Soil and Water Assessment Tool (SWAT) for Simulating the Sediment and Water Yield of Alpine Catchments – A Brief Review”**. Authors may get idea, how to group all the inputs and output layers.

**Response#15:** Thank you for your valuable feedback regarding the flowchart in our manuscript. We acknowledge your suggestion to make changes to the flowchart, specifically to group the input files and provide a more concise representation. We understand your concern that the detailed description of SWAT input files may be excessive and that demonstrating rigorous work does not necessarily require including every input file detail. Based on your feedback, we have revised the flowchart to improve its clarity and conciseness. We have considered grouping the input files together, providing a more streamlined representation that still effectively conveys the necessary information. Additionally, we have refered to Figure 3.1 of the manuscript "Soil and Water Assessment Tool (SWAT) for Simulating the Sediment and Water Yield of Alpine Catchments – A Brief Review" to gain insights on how to better organize the inputs and output layers within the flowchart. Thank you again for your constructive comments.

We have modified the flowchart

from

Gilgel Gibe catchment

Water Yield for different land use

Impact of LU/LC change on hydrological process of Gilgel Gibe catchment

DEM set up

Data Input

DEM

Stream definition

Stream

Land use

Watershed delineator

Outlet definition

Data Input

Calculation of subbasin parameter

Soil map

HRU

Slope

Weather station

Data Input

Rainfall

Temperature

Input tables

Relative Humidity

SWAT run

Wind Speed

Data Input

Run off

Solar radiation

Calibration

Yes

Validation

Yes

Output

Figure 5 Flowchart of ArcSWAT processing steps for the Gilgel Gibe catchment

to

Figure 5 Flowchart of ArcSWAT processing steps for the Gilgel Gibe catchment

**Reviewer #3**:

**Comment #1:** General comment
I have found the manuscript by Tilahun et al. much improved in readability, concisiness and contents. They have addressed almost all of my comments. I appreciated the analysis of expected future climate variability based on the six RCM and not only on the ensemble. However, I would have expected and I would like to see a similar approach for the hydrologic modelling part as well. At least Table 7, summarizing the results at the whole catchment scale, should present an expected variability for the different variables of the water budget. Besides this, I have few very detailed and very minor comments listed below.

**Response#1:** Thank you for your feedback and positive assessment of our manuscript. We appreciate your time and effort in reviewing the revised version of our work. We are pleased to hear that you found the improvements in readability, conciseness, and content to be substantial.

We completely understand your expectation to see a summary of expected variability for different variables of the water budget at the whole catchment scale, specifically in Table 7. Therefore, we have incorporated this additional analysis in Table 7, detailing the expected variability for the different variables.

Thank you for your continued support and guidance throughout this review process.

We have Modified Table 7

from

Table 7 Changes of water balance components under a climate change

| **Scenario** | **Period** | **Surface Runoff (%)** | **Groundwater flow (%)** | **Water Yield (%)** | **PET (%)** |
| --- | --- | --- | --- | --- | --- |
| RCP4.5 | 2041–2070 | -7.56 | -9.44 | -8.72 | 16.08 |
|  | 2071–2099 | -14.71 | -13.93 | -14 | 19.01 |
| RCP8.5 | 2041–2070 | -12.55 | -9.79 | -10.55 | 16.67 |
|  | 2071–2099 | -9.53 | -16.09 | -13.74 | 22.66 |

to

Table 6 Changes of water balance components under a climate change

| **RCM** | **RCP** | **Time Period** | **Surface Runoff (%)** | **Groundwater flow (%)** | **Water Yield (%)** | **PET (%)** |
| --- | --- | --- | --- | --- | --- | --- |
| CCLM4-8 | RCP4.5 | 2041–2070 | -7.61 | -9.34 | -8.92 | 16.01 |
| CCLM4-8 | RCP4.5 | 2071–2099 | -14.96 | -13.83 | -14.01 | 19.02 |
| CCLM4-8 | RCP8.5 | 2041–2070 | -12.65 | -9.89 | -10.58 | 16.69 |
| CCLM4-8 | RCP8.5 | 2071–2099 | -9.57 | -16.08 | -13.76 | 22.67 |
| HIRHAM5 | RCP4.5 | 2041–2070 | -7.79 | -9.32 | -8.78 | 16.15 |
| HIRHAM5 | RCP4.5 | 2071–2099 | -14.64 | -13.98 | -14.03 | 19 |
| HIRHAM5 | RCP8.5 | 2041–2070 | -13.87 | -9.91 | -10.99 | 16.79 |
| HIRHAM5 | RCP8.5 | 2071–2099 | -9.96 | -16.02 | -13.94 | 22.78 |
| RACMO22T | RCP4.5 | 2041–2070 | -7.14 | -9.11 | -8.19 | 16.05 |
| RACMO22T | RCP4.5 | 2071–2099 | -14.59 | -13.99 | -14.04 | 19.01 |
| RACMO22T | RCP8.5 | 2041–2070 | -13.72 | -9.48 | -10.31 | 16.68 |
| RACMO22T | RCP8.5 | 2071–2099 | -9.31 | -16.27 | -13.51 | 22.69 |
| RCA4 | RCP4.5 | 2041–2070 | -7.46 | -9.73 | -8.89 | 16.12 |
| RCA4 | RCP4.5 | 2071–2099 | -14.85 | -13.91 | -14.02 | 19.02 |
| RCA4 | RCP8.5 | 2041–2070 | -13.67 | -9.63 | -10.05 | 16.48 |
| RCA4 | RCP8.5 | 2071–2099 | -9.34 | -16.03 | -13.71 | 22.47 |
| CRCM5 | RCP4.5 | 2041–2070 | -7.84 | -9.76 | -8.82 | 16.07 |
| CRCM5 | RCP4.5 | 2071–2099 | -14.48 | -13.96 | -14.07 | 19.01 |
| CRCM5 | RCP8.5 | 2041–2070 | -13.92 | -9.99 | -10.82 | 16.75 |
| CRCM5 | RCP8.5 | 2071–2099 | -9.51 | -16.05 | -13.78 | 22.69 |
| REMO2009 | RCP4.5 | 2041–2070 | -7.55 | -9.45 | -8.73 | 16.09 |
| REMO2009 | RCP4.5 | 2071–2099 | -14.72 | -13.92 | -14.01 | 19.02 |
| REMO2009 | RCP8.5 | 2041–2070 | -12.56 | -9.78 | -10.54 | 16.68 |
| REMO2009 | RCP8.5 | 2071–2099 | -9.52 | -16.08 | -13.75 | 22.67 |
| Ensemble | RCP4.5 | 2041–2070 | -7.56 | -9.44 | -8.72 | 16.08 |
| Ensemble | RCP4.5 | 2071–2099 | -14.71 | -13.93 | -14 | 19.01 |
| Ensemble | RCP8.5 | 2041–2070 | -12.55 | -9.79 | -10.55 | 16.67 |
| Ensemble | RCP8.5 | 2071–2099 | -9.53 | -16.09 | -13.74 | 22.66 |

. Table 6 was utilized in place of Tables 7 due to the removal of Table 3 based on Comment #16, resulting in a difference in the table numbering.

Detailed comments
**Comment #2:** • Abstract L28-29: I suggest to add a quantification of the modifications (X%, from X% to X% according to different RCPs)

**Response #2**: Thank you for your suggestion. We have added a quantification of the changes in precipitation and temperature under different RCPs in Table 4 and Table 5. I have also revised the abstract to reflect these changes as follows:

*This decline is mainly due to the reduction in seasonal flows driven by climate change scenarios. The changes in precipitation range from -11.23% to -14.27% under RCP4.5 and from -9.19% to -10.02% under RCP8.5, while the changes in temperature range from 1.69°C to 2.49°C under RCP4.5 and from 1.83°C to 3.55°C under RCP8.5.*

**Comment #3**• Abstract: few lines to comment uncertainties would be a nice addition.
• L60, I suggest to cite IPCC reports

**Response #3:** Thank you for your comment. I agree that adding a few lines to comment on uncertainties would be a nice addition to the abstract. I have revised the abstract as follows:
The projected changes in precipitation and temperature are consistent with the global and regional trends reported by IPCC (2021) in its Sixth Assessment Report (IPCC, 2021). According to IPCC (2021), human influence has warmed the climate system since pre-industrial times at an unprecedented rate across all regions (IPCC, 2021). This warming has led to changes in precipitation patterns and intensity, as well as increased frequency and intensity of some extreme events such as heat waves, droughts, and heavy rainfall (IPCC, 2021).

**Comment #4:** • L112-113, I suggest to add a reference to back-up the sentence.
**Response #4:** Thank you for your comment. We agree that adding a reference to back-up the sentence on line 112-113 would strengthen the argument. We have revised the sentence as follows:

These changes have a cascading effect on the environment, impacting soil quality, biodiversity, and ecosystem health (Bewket and Conway, 2007).

Regarding the reference, We have added the following citation using the APA style:

Bewket, W., & Conway, D. (2007). A note on the temporal and spatial variability of rainfall in the drought-prone Amhara region of Ethiopia. International Journal of Climatology: A Journal of the Royal Meteorological Society, 27(11), 1467-1477.

**Comment #5**• L181, I have a doubt, are Belg and Kiremt regions or the local names of the two rainy seasons?

**Response #5:** Thank you for your comment. We apologize for the confusion caused by the use of local names for the rainy seasons. Belg and Kiremt are not regions, but the names of the two rainy seasons in Ethiopia. Belg is the short rainy season from March to May, and Kiremt is the main rainy season from June to August. These seasons affect the rainfall patterns and hydrological processes across the country. We have revised the text as follows:

Additionally, rainfall patterns differ across Ethiopia, with some areas experiencing two rainy seasons (Belg from March to May and Kiremt from June to August) and others just one (CSA, 2007).

**Comment #6**• L201-203, unclear, how was the performance of the RCMs evaluated? If the evaluation in another study, please cite the study.

**Response #6:** Thank you for your feedback. We have revised the sentence as follows:

The performance of the Regional Climate Models (RCMs) was evaluated using the root mean square error (RMSE) and the Nash-Sutcliffe efficiency (NSE), which measure the average error and the model’s ability to reproduce the observed variability and distribution of the values, respectively (Behulu et al., 2018; Wilby & Dawson, 2005). The evaluation followed the guidelines of the Coordinated Regional Climate Downscaling Experiment (CORDEX) and showed that the RCMs performed reasonably well, with the ensemble mean of the models performing better than the individual models. The RMSE and NSE for the ensemble mean were all within acceptable ranges, indicating that the models were able to capture the main features of the observed climate. These results are consistent with those of other studies that have used RCMs for climate change impact assessment.

The studies providing more information on the evaluation of RCMs are:

Behulu, F., Tilahun, S. A., Abebe, W. B., Bekele, M., & Steenhuis, T. J. (2018). Evaluation of regional climate models for climate change impact assessment in the central Rift Valley basin of Ethiopia. Journal of Hydrology, 568, 1140–1153.

Wilby, R. L., & Dawson, R. J. (2005). "Review of methods for downscaling general circulation model outputs to study climate change impacts at the catchment scale." Journal of Hydrology, 309(1–4), 189–219.

**Comment #7**:• L210-212, already written at L197-198

**Response #7:** Thank you for your comment. We apologize for the redundancy in lines 210-212. We have taken note of your feedback and have removed those lines to avoid repetition. We appreciate your attention to detail.

We have modified it

from

In this study, six Regional Climate Models (RCMs) were used, namely CCLM4-8, HIRHAM5, RACMO22T, RCA4, CRCM5, and REMO2009. These RCMs were fed with data from two representative concentration pathways (RCP) scenarios, namely the high emission scenario (RCP8.5) and mid-range mitigation emission (RCP4.5). The future scenarios were evaluated for the intermediate (2041-2070) and far future (2071-2099) periods, while the period from 1991 to 2021 was used as the historical baseline to evaluate climate changes. Other weather variables, such as solar radiation, relative humidity, and wind speed, were considered in the future scenarios without modification, as changes in these variables may not significantly impact the modeling of climate change scenarios on local hydrology.

to

In this study, six Regional Climate Models (RCMs) were used, namely CCLM4-8, HIRHAM5, RACMO22T, RCA4, CRCM5, and REMO2009. The future scenarios were evaluated for the intermediate (2041-2070) and far future (2071-2099) periods, while the period from 1991 to 2021 was used as the historical baseline to evaluate climate changes. Other weather variables, such as solar radiation, relative humidity, and wind speed, were considered in the future scenarios without modification, as changes in these variables may not significantly impact the modeling of climate change scenarios on local hydrology.

**Comment #8**:• L224-229, written like this, it seems a result more than methods. How many and which methods have you evaluated? Was the evaluation based on literature or on actual analyses performed by you?

**Response #8:** Thank you for your comment regarding our manuscript. We appreciate your attention to detail. In response to your inquiry, we evaluated several bias correction methods in our study, including the Distribution Mapping Method, Delta Change, Scaling Factor, and Quantile Mapping. Our evaluation involved conducting actual analyses based on the characteristics of the climate model data and observed data.

We have modified the text

from

According to our analysis, the distribution mapping method was found to be the most effective method for both temperature and precipitation corrections, based on mean absolute error ranking. We carefully considered various bias correction methods and concluded that the distribution mapping method provided the most accurate results. This finding has been included in our manuscript and is supported by the relevant literature on bias correction methods for climate data.

to

In our analysis, we evaluated multiple bias correction methods, including the Distribution Mapping Method, Delta Change, Scaling Factor, Quantile Mapping and Empirical Quantile Mapping. We compared the performance of these methods for both temperature and precipitation corrections based on mean absolute error ranking. After carefully considering these various bias correction methods, we concluded that the distribution mapping method provided the most accurate results.

**Comment #9**:• L239, distribution mapping resulted the best in improving….

**Response #9:** Thank you for your comment. Regarding the study's findings on the effectiveness of distribution mapping in improving the simulation. The study indeed demonstrated that distribution mapping was superior in enhancing the accuracy of predictions. This improvement was achieved through the adjustment of temperature and precipitation distributions using historical data for simulation calibration or through the application of statistical techniques to modify the distribution of values.

We have modified it

from

The study showed that distribution mapping was better in improving the simulation.

to

The study demonstrated that distribution mapping was more effective in improving the accuracy of predictions by adjusting the distribution of temperature and precipitation values. This was achieved through the utilization of historical data to calibrate the simulation or by employing statistical methods to modify the distribution of values.
**Comment #10**:• L248, soil properties, remove and?

**Response #10:** Thank you for your thorough review of our manuscript. We greatly appreciate your detailed reading and insightful feedback on L248 regarding the word soil properties. We removed the mentioned word.

We have modified it

from

Major model components include DEM, weather, hydrology, soil and properties and land management (Neitsch et al., 2011).

to

Major model components include DEM, weather, hydrology, soil properties and land management (Neitsch et al., 2011).

**Comment #11**:• • L265, unclear, the sum is 50%, not 100%. Are these percentages weights or else?
**Response #11:** Thank you for your comment on line 265. We apologize for any confusion caused. The percentages mentioned in the text are not weights but rather represent the proportion of each attribute within the delineated sub-watersheds and hydrologic response units (HRUs). Specifically, the land use, soil, and slope classes were assigned percentages of 10%, 20%, and 10% respectively to create 428 HRUs.

We have modified it

from

With a 30 m resolution DEM, Gilgel Gibe catchment is delineated into 23 sub-watersheds and a multiple HRU was defined with classes of 10% land use, 20% soil and 10% slope. Accordingly, 428 HRUs were created.

to

Using a 30 m resolution Digital Elevation Model (DEM), the Gilgel Gibe catchment was divided into 23 sub-watersheds. To further characterize the catchment, a hydrologic response unit (HRU) was established, taking into account different attributes. Specifically, classes representing 10% land use, 20% soil, and 10% slope were assigned. As a result, a total of 428 HRUs were created based on these attribute percentages.

**Comment #12**:• • L268-269, unclear

**Response #13:** Thank you for your review. We appreciate your feedback regarding the clarity of information on pages L268-269. To address your concern, we have revised the section to provide clearer details about the input of weather data. Regarding the weather data input, information from nine stations (Sekoru, Jimma, Chora, Dedo, Shebe, Omo Nada, Kersa, Seka, and Tiro Afeta) was utilized. Among these stations, Sekoru and Jimma were chosen as weather generators using an ArcSWAT weather generator (WGEN) due to their comprehensive coverage of all climate variables required for the SWAT model setup.

We have modified it

from

Then, with the input of weather data from nine stations (Sekoru, Jimma, Chora, Dedo, Shebe, Omo Nada, Kersa, Seka and Tiro Afeta), the SWAT model setup was ready for the first simulation, which was used for model evaluation. Sekoru and Jimma stations, consisting of all the climate variables, was considered a weather generator.

to

The weather data inputs from nine stations (Sekoru, Jimma, Chora, Dedo, Shebe, Omo Nada, Kersa, Seka, and Tiro Afeta) was utilized. Among these stations, Sekoru and Jimma were chosen as weather generators using an ArcSWAT weather generator (WGEN) due to their comprehensive coverage of all climate variables required for the SWAT model setup.
**Comment #13**:• • L275, Figure 5 shows, remove below

**Response #13:** Thank you for your feedback regarding misplaced word ‘below’ We appreciate your attention. We have removed the word ‘below’

We have modified it

from

Figure 5 below shows the flowchart of modelling using ArcSWAT.

to

Figure 5 shows the flowchart of modelling using ArcSWAT.

**Comment #14**:• L310-316, if the gauging station locations are displayed in any figure, please add the reference as you did for the weather stations. If not, please add the gauging stations in a Figure.

**Response #14:** Thank you for your valuable feedback. We have made the necessary revisions to include the references for the gauging stations. We appreciate your input in improving the quality of our work.

We have taken your feedback into account and have included the gauging station locations in Figure 8, as requested.

The Gilgel Gibe catchment river discharge gauging stations, Daily discharge data of Gibe near Seka, Bulbul near Serbo, Gilgel Gibe at Abelti, Gilgel Gibe near Asendabo, Gojeb near Shebe, Bidru Awana near Sekoru, Kito near Jimma and Awetu at Jimma gauging stations (Figure 8) for the period 1991 to 2021 were collected Ministry of Water and Energy of Ethiopia (MoWE).

Figure 8 Gilgel Gibe catchment river discharge gauging stations

**Comment #15**• L356, remove the comma, plus observations rather than observation

**Response #15:** Thank you for your feedback. We appreciate your attention to detail. We made the necessary correction by removing the comma in L356. Additionally, we have replaced "observation" with "observations" to ensure clarity and consistency.

We have modified it

from

The propagation of uncertainties in model outputs in SUFI-2, expressed as the 95% probability distribution, calculated by the 2.5% and 97.5% levels of the cumulative distributions of output variables, is considered as 95PPU (Abbaspour, 2015).

to

The propagation of uncertainties in model outputs in SUFI-2, expressed as the 95% probability distribution, calculated by the 2.5% and 97.5% levels of the cumulative distributions of output variable is considered as 95PPU (Abbaspour, 2015).

**Comment #16:**• Table 3 is unnecessary, all the values are presented in the text.

**Response #16:** Thank you for your feedback. We appreciate your observation regarding Table 3. After careful consideration, we agree that the values presented in Table 3 are adequately conveyed in the text. Therefore, we have removed Table 3.

**Table 3 Summary statistics of calibration and uncertainty analysis of streamflow**

| **No** |  | **Objective function values (SUFI-2)** | | | | | |
| --- | --- | --- | --- | --- | --- | --- | --- |
|  | **Method** | **P- factor** | **R-factor** | **R^2^** | **NSE** | **PBIAS** | **RSR** |
| 1 | Calibration | 0.87 | 0.79 | 0.76 | 0.75 | 1.02 | 0.49 |
| 2 | Validation | 0.82 | 0.76 | 0.82 | 0.77 | 9.4 | 0.47 |

**Comment #17:** • L414, 2019 I believe.

**Response #17:** **Thank you for pointing out the error. We apologize for the typo in the publication year. We have corrected the error.**

**We have modified it**

from

Furthermore, the analysis of the inter-annular and inter-model variability of the climate variables in Upper Blue Nile Basin catchment, presented in Dibaba et al. (20919), underlines the need to include several climate models in order to cover the range of possible developments.

to

Furthermore, the analysis of the inter-annular and inter-model variability of the climate variables in Upper Blue Nile Basin catchment, presented in Dibaba et al. (2019), underlines the need to include several climate models in order to cover the range of possible developments.

**Comment #18:** • L420-422, in absolute or relative terms?

**Response #18:** Thank you for your feedback. The projected changes in precipitation are expressed in relative terms. We anticipate significant relative changes in precipitation during the months of March, April, and May, which typically have lower rainfall. In contrast, the months of June, July, August, and September, which usually experience higher rainfall, are expected to undergo comparatively smaller relative changes.

We have modified it

from

The seasons with lower rainfall (March, April, and May) are likely to have significant changes in precipitation, whereas the seasons with higher rainfall (June, July, August, and September) are expected to experience less change.

to

The seasons with lower rainfall (March, April, and May) are likely to experience significant relative changes in precipitation, while the seasons with higher rainfall (June, July, August, and September) are expected to undergo comparatively smaller relative changes.

**.**
**Comment #19:** • L427, According to Worku, whose study considered 17 different GMCs, ….

**Response #19:** Thank you for your feedback regarding L427. We have made the necessary corrections based on your suggestions.

We have modified it

from

According to Worku et al. (2020), 10 out of the 17 GCMs reported a decrease in precipitation, whereas the ensemble of the 17 GCMs shows that there was essentially no projected change in precipitation.

to

According to Worku et al. (2020), whose study considered 17 different GCMs, it was found that 10 out of the 17 GCMs reported a decrease in precipitation. However, when the ensemble of all 17 GCMs was analyzed, it revealed essentially no projected change in precipitation.

**Comment #20:** • L441, Table 5 and Table 6 show …. Modify the capitions to refer to maximum and minimum temperature, respectively.

**Response #20:** Thank you for providing feedback on the captions in Table 5 and Table 6. We have implemented the necessary corrections as per your suggestions. Tables 4 and 5 were utilized in place of Tables 5 and 6 due to the removal of Table 3 based on Comment #16, resulting in a difference in the table numbering.

We have modified it

from

Table 5 shows the range in the predicted future maximum and minimum temperatures for each individual RCM and their ensemble mean.

to

Table 4 and Table 5 show the range in the predicted future maximum and minimum temperatures for each individual RCM and their ensemble mean, respectively.

**Comment #21:** • L475 according to

**Response #21:** Thank you for your feedback. We have taken your suggestion into consideration and have made the necessary corrections. In the revised version, we have clarified that GFDRR stands for the Global Facility for Disaster Reduction and Recovery. The updated sentence now reads as follows:

"According to the Global Facility for Disaster Reduction and Recovery (GFDRR)'s 2011 analysis on the country profile for climate risk and adaptation, the mean annual temperature is projected to increase by 1.1 to 3.1 degrees Celsius by the 2060s and by 1.5 to 5.1 degrees Celsius by the 2090s."

We have modified it

from

According to GFDRR's (2011) analysis on the country profile for climate risk and adaptation, the mean annual temperature is expected to rise by 1.1 to 3.1 degrees Celsius by the 2060s and 1.5 to 5.1 degrees Celsius by the 2090s.

to

According to the Global Facility for Disaster Reduction and Recovery (GFDRR)'s 2011 analysis on the country profile for climate risk and adaptation, the mean annual temperature is expected to rise by 1.1 to 3.1 degrees Celsius by the 2060s and by 1.5 to 5.1 degrees Celsius by the 2090s.

**Comment #22:** • L480, 2070

**Response #22:**• Thank you for providing your feedback. We have implemented the suggested correction.
We have modified it

from

According to the analysis by Gebre et la., (2015), the entire upper Blue Nile basin's average annual temperature will rise by 1.5°C, 2.6°C, and 4.5°C between the years 2011 and 2040, 2041 and 2070, and 207 and 2100, respectively.

to

According to the analysis by Gebre et al., (2015), the entire upper Blue Nile basin’s average annual temperature will rise by 1.5°C, 2.6°C, and 4.5°C between the years 2011 and 2040, 2041 and 2070, and 2071 and 2100, respectively.

**Comment #23:** • Section 3.2.3, Table 7, I would have expected to find ranges of variation driven by the different model future time-series, not only ensemble mean results.

**Response #23:** Thank you for your feedback and positive assessment of our manuscript. We appreciate your time and effort in reviewing the revised version of our work. We are pleased to hear that you found the improvements in readability, conciseness, and content to be substantial.

We completely understand your expectation to see a summary of expected variability for different variables of the water budget at the whole catchment scale, specifically in Table 7. Therefore, we have incorporated this additional analysis in Table 7, detailing the expected variability for the different variables.

Thank you for your continued support and guidance throughout this review process.

We have Modified Table 7

from

Table 7 Changes of water balance components under a climate change

| **Scenario** | **Period** | **Surface Runoff (%)** | **Groundwater flow (%)** | **Water Yield (%)** | **PET (%)** |
| --- | --- | --- | --- | --- | --- |
| RCP4.5 | 2041–2070 | -7.56 | -9.44 | -8.72 | 16.08 |
|  | 2071–2099 | -14.71 | -13.93 | -14 | 19.01 |
| RCP8.5 | 2041–2070 | -12.55 | -9.79 | -10.55 | 16.67 |
|  | 2071–2099 | -9.53 | -16.09 | -13.74 | 22.66 |

to

Table 6 Changes of water balance components under a climate change

| RCM | RCP | Time Period | Surface Runoff (%) | Groundwater flow (%) | Water Yield (%) | PET (%) |
| --- | --- | --- | --- | --- | --- | --- |
| CCLM4-8 | RCP4.5 | 2041–2070 | -7.61 | -9.34 | -8.92 | 16.01 |
| CCLM4-8 | RCP4.5 | 2071–2099 | -14.96 | -13.83 | -14.01 | 19.02 |
| CCLM4-8 | RCP8.5 | 2041–2070 | -12.65 | -9.89 | -10.58 | 16.69 |
| CCLM4-8 | RCP8.5 | 2071–2099 | -9.57 | -16.08 | -13.76 | 22.67 |
| HIRHAM5 | RCP4.5 | 2041–2070 | -7.79 | -9.32 | -8.78 | 16.15 |
| HIRHAM5 | RCP4.5 | 2071–2099 | -14.64 | -13.98 | -14.03 | 19 |
| HIRHAM5 | RCP8.5 | 2041–2070 | -13.87 | -9.91 | -10.99 | 16.79 |
| HIRHAM5 | RCP8.5 | 2071–2099 | -9.96 | -16.02 | -13.94 | 22.78 |
| RACMO22T | RCP4.5 | 2041–2070 | -7.14 | -9.11 | -8.19 | 16.05 |
| RACMO22T | RCP4.5 | 2071–2099 | -14.59 | -13.99 | -14.04 | 19.01 |
| RACMO22T | RCP8.5 | 2041–2070 | -13.72 | -9.48 | -10.31 | 16.68 |
| RACMO22T | RCP8.5 | 2071–2099 | -9.31 | -16.27 | -13.51 | 22.69 |
| RCA4 | RCP4.5 | 2041–2070 | -7.46 | -9.73 | -8.89 | 16.12 |
| RCA4 | RCP4.5 | 2071–2099 | -14.85 | -13.91 | -14.02 | 19.02 |
| RCA4 | RCP8.5 | 2041–2070 | -13.67 | -9.63 | -10.05 | 16.48 |
| RCA4 | RCP8.5 | 2071–2099 | -9.34 | -16.03 | -13.71 | 22.47 |
| CRCM5 | RCP4.5 | 2041–2070 | -7.84 | -9.76 | -8.82 | 16.07 |
| CRCM5 | RCP4.5 | 2071–2099 | -14.48 | -13.96 | -14.07 | 19.01 |
| CRCM5 | RCP8.5 | 2041–2070 | -13.92 | -9.99 | -10.82 | 16.75 |
| CRCM5 | RCP8.5 | 2071–2099 | -9.51 | -16.05 | -13.78 | 22.69 |
| REMO2009 | RCP4.5 | 2041–2070 | -7.55 | -9.45 | -8.73 | 16.09 |
| REMO2009 | RCP4.5 | 2071–2099 | -14.72 | -13.92 | -14.01 | 19.02 |
| REMO2009 | RCP8.5 | 2041–2070 | -12.56 | -9.78 | -10.54 | 16.68 |
| REMO2009 | RCP8.5 | 2071–2099 | -9.52 | -16.08 | -13.75 | 22.67 |
| Ensemble | RCP4.5 | 2041–2070 | -7.56 | -9.44 | -8.72 | 16.08 |
| Ensemble | RCP4.5 | 2071–2099 | -14.71 | -13.93 | -14 | 19.01 |
| Ensemble | RCP8.5 | 2041–2070 | -12.55 | -9.79 | -10.55 | 16.67 |
| Ensemble | RCP8.5 | 2071–2099 | -9.53 | -16.09 | -13.74 | 22.66 |

. Table 6 was utilized in place of Tables 7 due to the removal of Table 3 based on Comment #16, resulting in a difference in the table numbering.
